# Single‐Cell Immune Profiling Reveals Neutrophils Promote Myasthenia Gravis Exacerbation Through BAFF Secretion

**DOI:** 10.1002/advs.202509260

**Published:** 2025-09-25

**Authors:** Zhaoxu Zhang, Jingya Dong, Mengyuan Qiu, Jie Bai, Xufeng Cheng, Xiaodong Song

**Affiliations:** ^1^ Department of Neurology Peking University People's Hospital Beijing 100044 China; ^2^ Program in Integrated Biology and Medicine Duke–NUS Medical School, Singapore Singapore 169857 Singapore; ^3^ Department of Infectious Diseases The First Affiliated Hospital of Chongqing Medical University Chongqing 400016 China; ^4^ Department of Neurology Beijing Jishuitan Hospital Capital Medical University Beijing 100035 China; ^5^ Department of Neurology The First Affiliated Hospital of Chongqing Medical University Chongqing 400016 China

**Keywords:** B‐cell activating factor, myasthenia gravis, neutrophils, single‐cell RNA sequencing

## Abstract

Neutrophils play a critical role in the pathogenesis of autoimmune diseases, including myasthenia gravis (MG), but their specific function in MG exacerbations remains unclear. This study utilizes single‐cell RNA sequencing (scRNA‐seq) of bone marrow and peripheral blood from MG patients during acute exacerbations, combined with experimental autoimmune myasthenia gravis (EAMG) mouse models and clinical cohort analyses, to investigate the potential involvement of a neutrophil–B‐cell activating factor (BAFF) –plasma cell axis. The results reveal that, during MG acute exacerbation, bone‐marrow neutrophils exhibit significantly enhanced maturation. Upon migration to the peripheral blood, these neutrophils secrete increased amounts of BAFF, further promoting pathological B‐cell differentiation and plasma cell activation. Moreover, gene knockout models and serum cytokine analyses reveal that the IFN‐γ signaling pathway is a key driver of this excessive BAFF secretion, supporting the existence of a neutrophil–BAFF–plasma cell interaction relevant to MG exacerbation. Clinical data analysis shows that MG patients with high baseline neutrophil levels derive greater benefit from treatment with the BAFF/APRIL dual‐target inhibitor telitacicept. Collectively, this study reveals the mechanistic link between neutrophil activation and MG exacerbation, providing insights that may inform precision‐targeted immunotherapy.

## Graphical abstract

Single‐cell transcriptomics and clinical data show that during myasthenia gravis (MG) exacerbation, bone marrow mature neutrophils expand and enter blood, with parallel increase of B‐cell activating factor (BAFF)–positive neutrophils. Experimental autoimmune myasthenia gravis (EAMG) confirms a pathogenic interferon‐γ (IFN‐γ)–neutrophil–BAFF axis. Patients with higher baseline neutrophils respond better to BAFF‐targeted therapy.

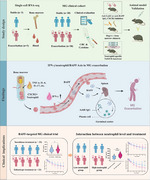

## 1 Introduction

Myasthenia Gravis (MG) is an autoimmune disease characterized by fluctuating skeletal muscle weakness and pathological fatigue. Its core pathological mechanism arises from the abnormal production of autoantibodies targeting key proteins of the post‐synaptic membrane at the neuromuscular junction (NMJ).^[^ ^1^, ^2^ ^]^ In approximately 80%–85% of cases, anti‐acetylcholine receptor (AChR) antibodies block acetylcholine binding sites or activate complement‐mediated membrane attack complexes, leading to the destruction of the post‐synaptic membrane and loss of signal transmission efficiency.^[^ ^3^ ^]^ In the remaining cases, the antibodies are related to muscle‐specific kinase or low‐density lipoprotein receptor‐related protein 4.^[^ ^3^ ^]^ Epidemiological studies show that the global prevalence of MG is approximately 12.4 cases per million people, with about 10% of patients exhibiting treatment resistance to standard immunomodulatory therapy, posing a significant clinical management challenge.^[^ ^4^, ^5^ ^]^ The disease course of MG follows a heterogeneous pattern of chronic progression and episodic exacerbations. During acute exacerbation, a rapid decline in NMJ signal transmission efficiency can rapidly progress to a life‐threatening myasthenic crisis, clinically manifested as acute respiratory muscle paralysis or bulbar muscle dysfunction, usually requiring urgent mechanical ventilation support.^[^ ^6^ ^]^ Notably, exposure to infectious pathogens, surgical interventions related to thymic hyperplasia, pregnancy‐related immune adaptations, and improper tapering of immunosuppressants may increase the risk of MG exacerbations.^[^ ^7^, ^8^, ^9^ ^]^ However, the underlying immune molecular mechanisms of MG acute exacerbations remain incompletely elucidated.

Although the main cell types involved in the pathogenesis of MG are known, the key cell subsets, their transcriptomic characteristics, and how their interactions contribute to the mechanisms of acute exacerbation in MG remain unclear. Anti‐AChR antibodies serve as diagnostic and disease classification markers for MG patients, but cannot reflect or predict the occurrence of acute exacerbations.^[^ ^7^ ^]^ Conventional methods for cell subset detection and sorting, such as flow cytometry, have certain limitations and cannot fully capture and analyze the changes in the immune environment of patients during acute exacerbations of MG. Given that different cell types contribute to the disease in a specific manner and that complex signaling networks exist between cells, focusing solely on one or a few immune cells may obscure cellular heterogeneity and hinder the exploration of the mechanisms of acute exacerbation and the discovery of potential targets in MG. In recent years, single‐cell RNA sequencing (scRNA‐seq), with its unique advantage of single‐cell resolution, has become a milestone in technology for disease mechanism research. scRNA‐seq may deepen our understanding of disease mechanisms and provide valuable data that can help identify potential therapeutic targets and dysregulated signaling pathways in MG.^[^ ^10^ ^]^

In this study, we performed scRNA‐seq on bone marrow and peripheral blood immune cells from patients with acute exacerbation and stable MG, constructing the immune landscape of peripheral blood and bone marrow during acute exacerbation of MG for the first time. We explored the cell types that are pathogenic during this phase. Our findings revealed that neutrophils exhibited significant changes in both proportion and function in the bone marrow during acute exacerbation. Further validation using scRNA‐seq of peripheral blood from patients with acute exacerbation, together with experiments in the experimental autoimmune myasthenia gravis (EAMG) mouse model, suggested that neutrophils may promote plasma cell responses by releasing B‐cell activating factor (BAFF), thereby contributing to acute exacerbation in MG. Additionally, a clinical trial studying the treatment of MG patients with the BAFF/APRIL (a proliferation‐inducing ligand) dual‐target inhibitor telitacicept further supports this hypothesis. Our study provides new insights into the role of neutrophils during acute exacerbation of MG, suggesting that inhibiting neutrophil‐derived BAFF secretion could serve as an effective targeted immunotherapy strategy, offering new treatment opportunities for MG patients.

## 2 Results

### 2.1 Single‐cell Transcriptomics Reveal Major Immune Cell Alterations in the Bone Marrow during Acute Exacerbation of MG

**Figure 1:**
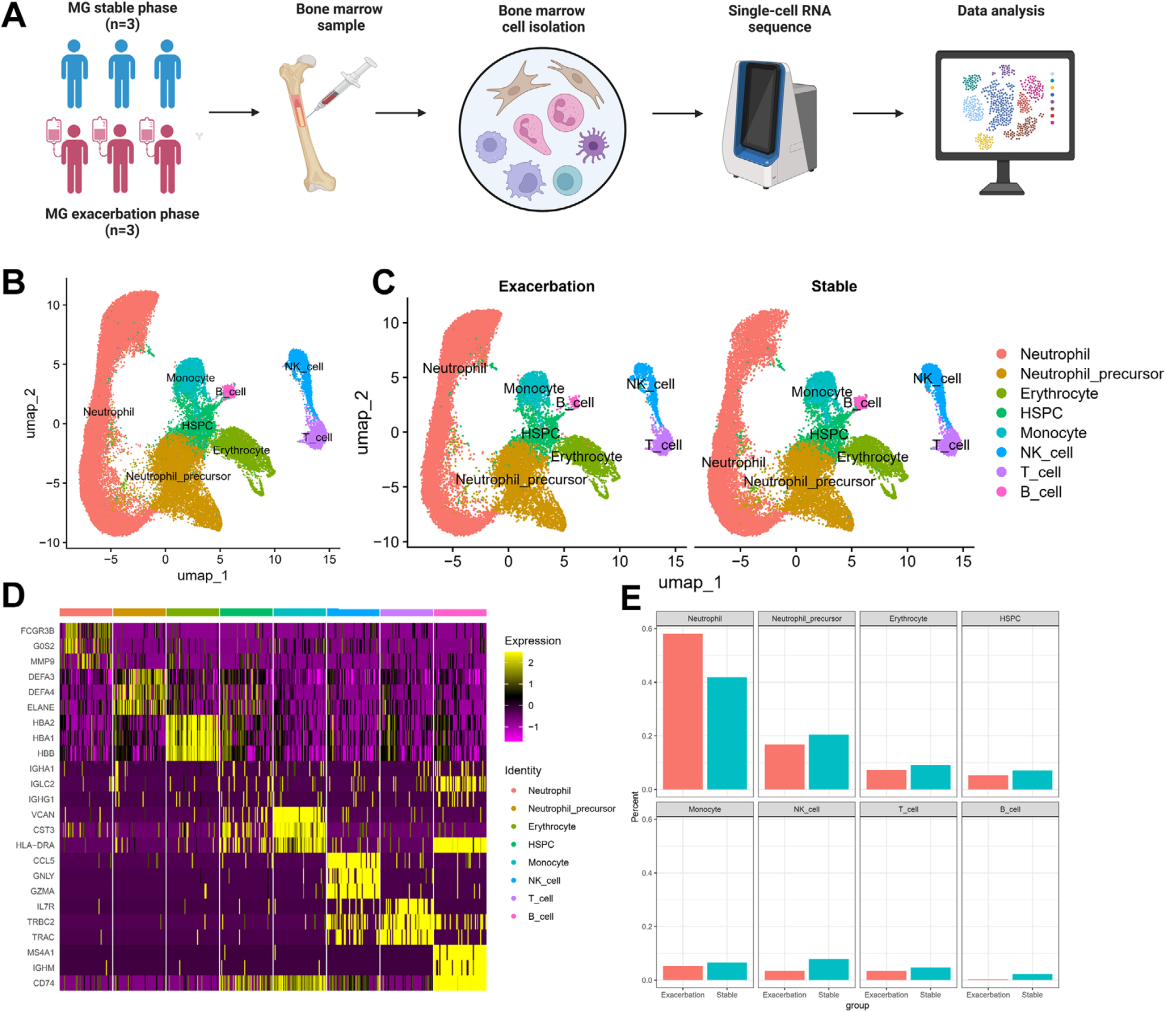
MG exacerbation bone marrow cell transcriptional atlas. A) Experimental design for scRNA‐seq analysis of bone marrow cells from MG patients at the stable phase (*n* = 3) and exacerbation phase (*n* = 3). B) UMAP plot of merged bone marrow cells. C) UMAP plot of merged bone marrow cells from the MG exacerbation phase (left) and stable phase (right). D) Heatmap showing the expression levels of the top three marker genes for the corresponding cell types in the bone marrow. E) Relative changes in the cell‐type proportions of bone marrow cells between the MG exacerbation phase (left) and stable phase (right). Abbreviations: MG, myasthenia gravis; UMAP, uniform manifold approximation and projection.

To comprehensively characterize immune alterations at the single‐cell level in the bone marrow of MG patients during acute exacerbation, we performed scRNA‐seq using the 10x Genomics Chromium platform on iliac crest bone marrow samples from three untreated MG patients in the stable phase and three age‐ and sex‐matched patients in the acute exacerbation phase. A total of 49 630 high‐quality single‐cell transcriptomes were obtained (**Figure** **1**A). Using unsupervised clustering combined with canonical cell markers, we classified the single‐cell transcriptomes into eight cell types, including neutrophils, neutrophil progenitors, hematopoietic stem and progenitor cells (HSPCs), B cells, monocytes, erythroid cells, T cells, and natural killer (NK) cells (Figure 1B,C). Cell types were identified based on known marker genes: S100A8 and S100A9 (neutrophils), AZU1, ELANE, and MKI67 (neutrophil progenitors), CD79A and MS4A1 (B cells), CD14 (monocytes), HBA1 and HBD (erythroid cells), CD3E and CD3D (T cells), and KLF1 and GNLY (NK cells) (Figure 1D; Figure S1A,B, Supporting Information). Cell proportion analysis revealed that neutrophils were the only bone marrow subset that increased during acute exacerbation compared with the stable phase (58.1% vs. 41.9%; Figure 1E, Figure S1C–E, Supporting Information). Given the limited sample size of this scRNA‐seq study, we focused on neutrophils—a pro‐inflammatory subset exhibiting an expansion in abundance during acute exacerbation—as a potential target for therapeutic intervention.

### 2.2 Mature Neutrophils are Increased in the Bone Marrow during Acute Exacerbation of MG

**Figure 2:**
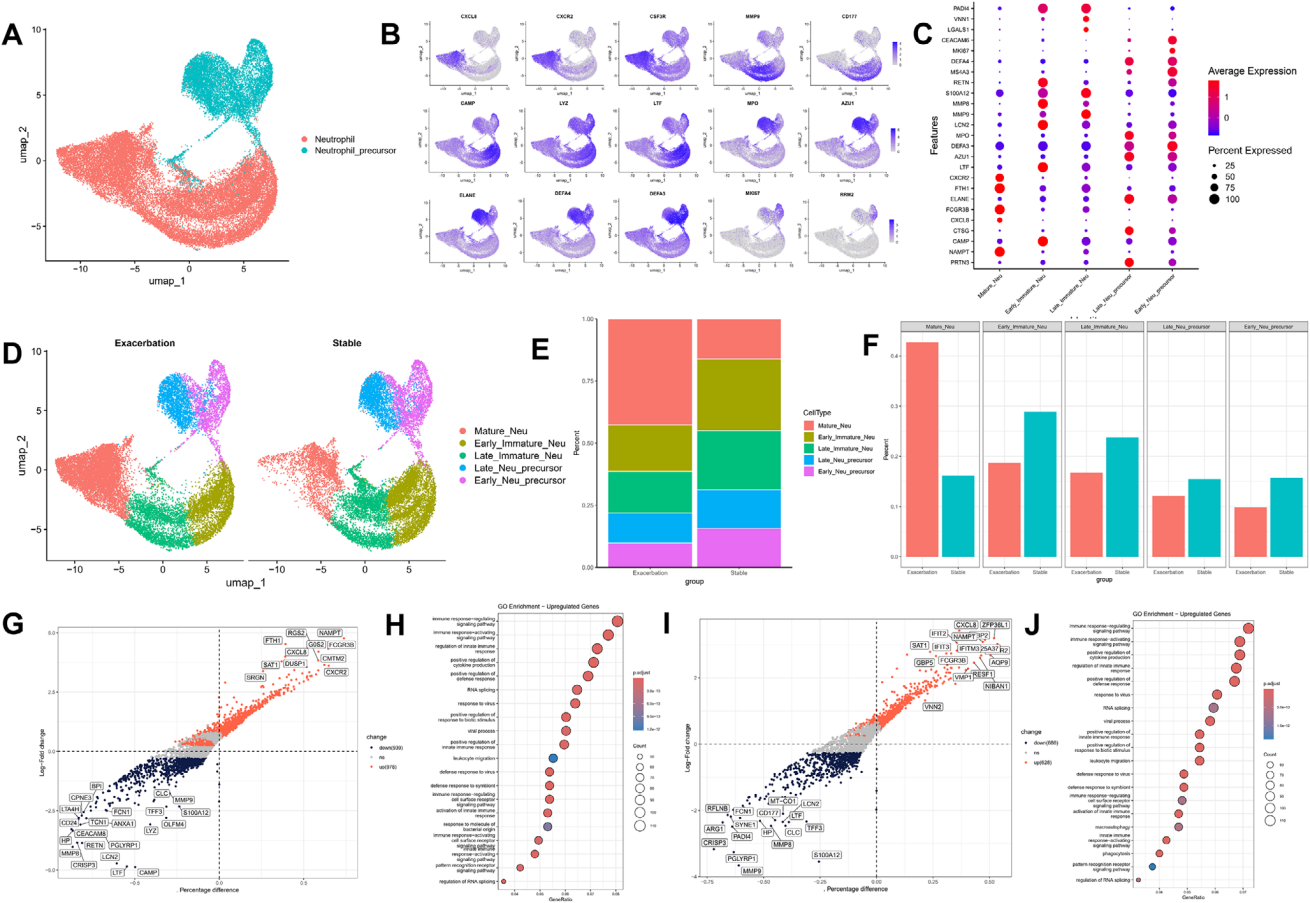
Exploration of differences in bone marrow neutrophils between the exacerbation and stable phase. A) UMAP plot of bone marrow neutrophils. B) Feature plot displaying the expression patterns of representative markers in bone marrow neutrophils. C) Dot plots displaying the top five marker gene expression profiles for different bone marrow neutrophil subgroups. D) UMAP plot of neutrophil subsets between the two groups, with color coding according to the subsets of manual annotation. E,F) The proportion of bone marrow neutrophil subsets in the two groups. G,I) DEGs between mature neutrophils and their immature counterparts (late immature in G, early immature in I), with red and blue dots representing upregulated and downregulated genes in mature neutrophils, respectively. H,J) GO enrichment bubble plots showing the top 20 GO terms of upregulated genes identified in the comparisons presented in (G) and (I), respectively. Abbreviations: MG, myasthenia gravis; DEGs, differentially expressed genes; UMAP, uniform manifold approximation and projection; GO, Gene Ontology.

To investigate changes in bone marrow neutrophils during the acute exacerbation phase of MG, we extracted and integrated neutrophils and neutrophil precursors for subsequent cell subset analysis (**Figure** **2**A). We visualized the data using two‐dimensional Uniform Manifold Approximation and Projection (UMAP) and performed unsupervised clustering in combination with canonical cell markers (Figure 2B,C). With reference to a previous study^[^ ^11^ ^]^ and the CellMarker 2.0 database, ^[^ ^12^ ^]^ neutrophils were classified into five developmental subsets: mature neutrophils (CXCR2⁺ CXCL8⁺ CSF3R⁺), late immature neutrophils (MMP9⁺ CD177⁺), early immature neutrophils (CAMP⁺ LTF⁺ LYZ⁺), late neutrophil precursors (MPO⁺ AZU1⁺ ELANE⁺), and early neutrophil precursors (DEFA3⁺ MKI67⁺ RRM2⁺) (Figure 2D; Figure S2A–D, Supporting Information).

Compared with the stable phase of MG, the proportions of neutrophil subsets changed significantly during the acute exacerbation phase, with the most notable alteration being a marked increase in mature neutrophils (42.7% vs. 16.1%, *p* = 0.042) (Figure 2E,F; Figure S2A,B, Supporting Information). To further investigate the functional differences between mature neutrophils and other neutrophil subsets, we compared the gene signatures and differentially expressed genes of mature neutrophils with those of late immature neutrophils (Figure 2G) and early immature neutrophils (Figure 2I). The results showed that, compared to immature neutrophils, mature neutrophils had significantly upregulated genes enriched in pathways related to innate immune response regulation (*p* < 0.001), immune response signaling (*p* < 0.001), antiviral response (*p* < 0.001), and leukocyte migration (*p* < 0.001) (Figure 2H,J). In contrast, the downregulated genes in mature neutrophils were mainly associated with oxidative stress (*p* < 0.001), oxidative phosphorylation (*p* < 0.001), ATP synthesis (*p* < 0.001), and other energy metabolism‐related pathways (*p* < 0.001) (Figure S2E‐J, Supporting Information).

Pseudotime analysis revealed that during the differentiation of neutrophil progenitors into mature neutrophils, genes related to immune regulation (such as FCGR3B and IFITM2) were gradually upregulated, while genes associated with early neutrophil functions (such as MMP8 and CRISP3) were progressively downregulated (Figure S3A,B, Supporting Information). No statistically significant difference was observed in the ratio of early to late neutrophil precursors between patients in the acute exacerbation and stable phases of MG (0.81 vs. 1.01, *p* > 0.05; Figure S3C, Supporting Information). At the cell‐cycle level, the G2/M + S fraction was numerically higher in the acute exacerbation group for both early and late neutrophil progenitors (88.1% vs. 83.1% and 37.8% vs. 34.9%, respectively); however, these differences were not statistically significant (both *p* > 0.05; Figure S3D,E, Supporting Information). Similarly, among HSPCs, the combined proportion of granulocyte–monocyte progenitors and neutrophil progenitors was higher in the acute exacerbation cohort than in the stable‐phase cohort (73.7% vs. 65.7%) without reaching statistical significance (*p* > 0.05; Figure S3F–H, Supporting Information).

### 2.3 Peripheral Blood Neutrophil Changes during Acute Exacerbation of MG

The progression of MG is often associated with dynamic increases in inflammatory markers such as the neutrophil‐to‐lymphocyte ratio (NLR), which has been recognized as a reliable marker for disease severity and a predictor of short‐term poor outcomes.^[^ ^13^, ^14^, ^15^, ^16^ ^]^ Our single‐cell transcriptome data from bone marrow samples during acute MG exacerbation revealed remodeling of the hematopoietic process, characterized by a significant increase in the proportion of mature neutrophils. We hypothesize that elevated blood neutrophil levels serve as an important inflammatory marker in acute exacerbation, reflecting hematopoietic remodeling and excessive immune activation.

**Figure 3:**
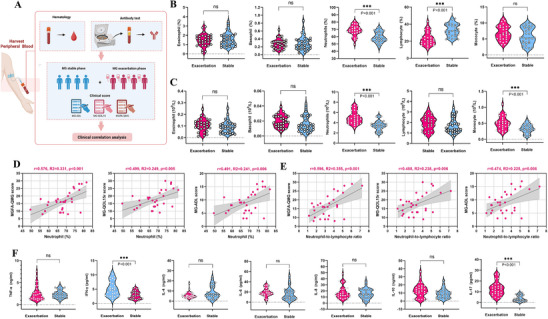
A retrospective study identifies neutrophil magnitude correlated with MG clinical severity. A) Schematic overview of the retrospective study design. B) Proportions of peripheral blood immune cells in MG exacerbation (*n* = 30) and stable (*n* = 30) groups. C) Absolute counts of peripheral blood immune cells in MG exacerbation (*n* = 30) and stable (*n* = 30) groups. D) Correlation between neutrophil proportions and MG‐related clinical scores (MGFA‐QMG, MG‐QOL15r, MG‐ADL) in the MG exacerbation group (*n* = 30). E) Correlation between NLR and MG clinical severity in the MG exacerbation group (*n* = 30). F) Serum levels of TNF‐α, IFN‐γ, IL‐4, IL‐6, IL‐8, IL‐10, and IL‐17 in the two groups. Abbreviations: MG, myasthenia gravis; NLR, neutrophil‐to‐lymphocyte ratio; MGFA‐QMG, Myasthenia Gravis Foundation of America Quantitative Myasthenia Gravis; MG‐QOL15r, Myasthenia Gravis Quality of Life 15‐item revised; MG‐ADL, Myasthenia Gravis Activities of Daily Living. ns, not significant; *p < 0.05; **p < 0.01; ***p < 0.001. *p*‐values were calculated using the Mann–Whitney U test B,C,F) or Spearman correlation analysis D,E). In all instances, *n* refers to the number in each group.

To assess neutrophil changes and their underlying mechanisms, we retrospectively analyzed complete blood counts and cytokine levels from 30 acute exacerbation MG patients and 30 stable‐phase MG patients (**Figure** **3**A). We excluded patients with concomitant infections to prevent confounding effects on neutrophil chemotaxis. Consistent with previous reports, patients with acute exacerbation of MG exhibited significantly higher neutrophil counts (4.76×10⁹ L^−1^ [IQR, 4.00–7.49] vs. 3.43×10⁹ L^−1^ [IQR, 2.88–5.43], *p* < 0.001) and neutrophil ratios (68.31% [IQR, 64.69–81.62] vs. 58.11% [IQR, 54.46–72.56], *p* < 0.001) compared with those in the stable phase (Figure 3B,C). Moreover, neutrophil ratio and NLR values were positively correlated with clinical scores (Figure 3D,E), suggesting their potential as dynamic biomarkers of disease progression in MG. Further analysis showed significantly elevated levels of IFN‐γ (4.70 pg mL^−1^ [IQR, 2.73–10.80] vs. 2.70 pg mL^−1^ [IQR, 1.70–5.50], *p* < 0.001) and IL‐17 (12.40 pg mL^−1^ [IQR, 8.35–27.10] vs. 2.55 pg mL^−1^ [IQR, 1.38–9.20], *p* < 0.001) in the peripheral blood of acute exacerbation MG patients compared with those in the stable phase (Figure 3F). Notably, both neutrophil ratio and NLR correlated positively with IL‐17 and anti‐AChR IgG, suggesting a potential link between IL‐17 and neutrophil elevation (Figure S4A, B).

### 2.4 Neutrophils Exhibited Significant Transcriptional Heterogeneity in the Peripheral Blood during Acute Exacerbation of MG

**Figure 4:**
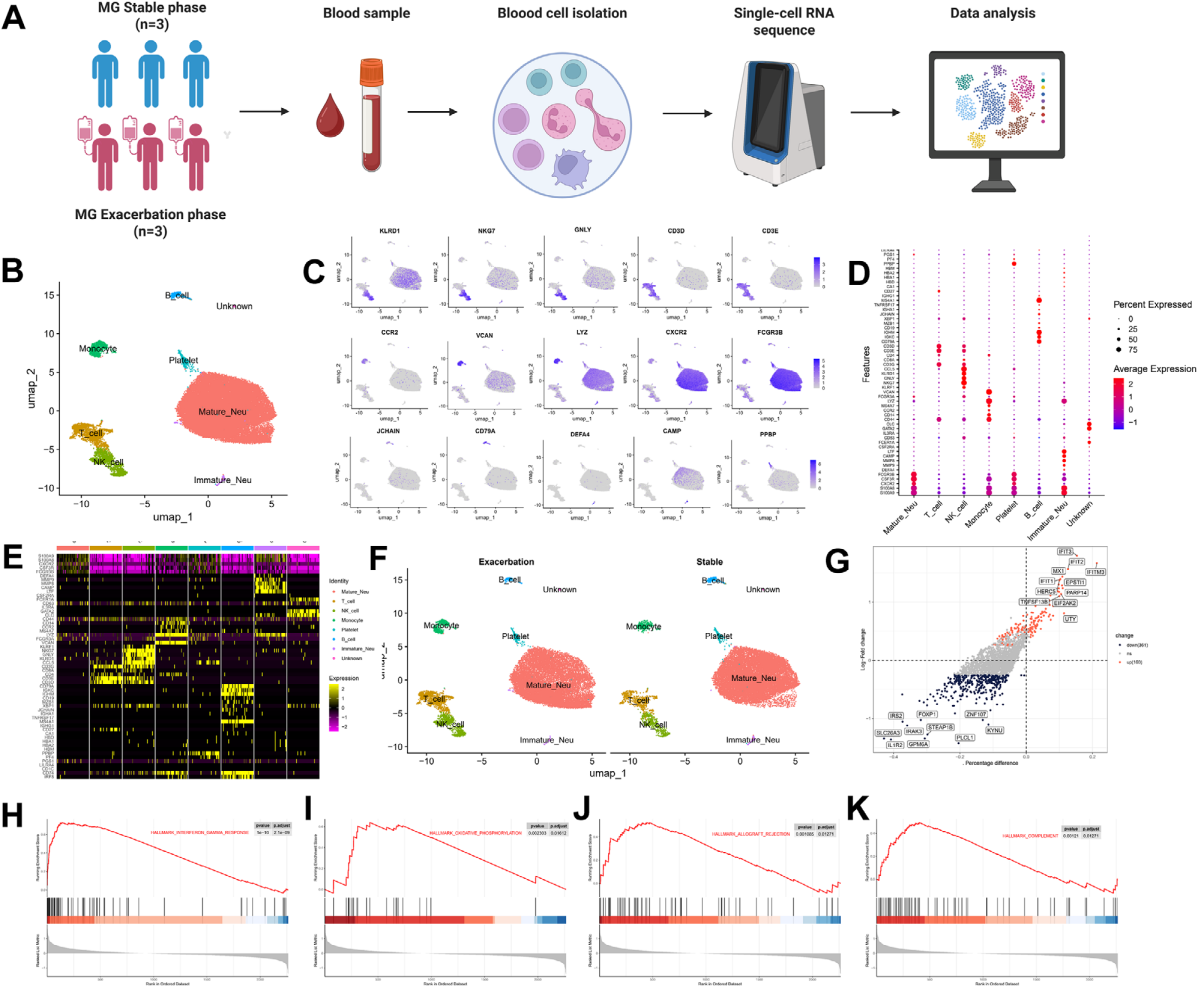
Transcriptional landscape of peripheral blood in MG exacerbation. A) Overview of the experimental design for scRNA‐seq analysis of peripheral blood cells from MG patients in stable *n* = 3) and exacerbation *n* = 3) phases. (B) UMAP representation of integrated peripheral blood cells. C) Feature plot showing the distribution of representative marker gene expression in peripheral blood cells. D,E) Marker gene expression profiles across peripheral blood cell types, visualized as a dot plot D) and heatmap E). F) UMAP plot of integrated peripheral blood cells from MG exacerbation (left) and stable (right) phases. G) Volcano plot of DEGs in mature neutrophils between MG exacerbation and stable phases, with red indicating upregulated and blue indicating downregulated genes. H–K) Hallmark GSEA analysis of upregulated genes identified in (G). Abbreviations: MG, myasthenia gravis; DEGs, differentially expressed genes; UMAP, uniform manifold approximation and projection; GSEA, Gene Set Enrichment Analysis.

To investigate the pathological regulatory mechanisms of neutrophils during the acute exacerbation of MG, we collected peripheral blood samples from the same six MG patients previously described (three in the acute exacerbation phase and three in the stable phase) to perform single‐cell RNA sequencing analysis (**Figure** **4**A). After strict quality control, 35 750 cells were obtained. Through UMAP clustering and verification with classical marker genes, seven immune cell types were identified: NK cells (KLRF1^+^ GNLY^+^ NKG7^+^), T cells (CD3D^+^ CD3E^+^), monocytes (CCR2^+^ VCAN^+^ LYZ^+^), mature neutrophils (CXCR2^+^ FCGR3B^+^), immature neutrophils (DEFA4^+^ CAMP^+^), B cells (MS4A1^+^ JCHAIN^+^), and platelets (PPBP^+^) (Figure 4B–F). Notably, mature neutrophils in the MG exacerbation group exhibited significant transcriptional heterogeneity, with differential gene analysis showing upregulation of genes such as TNFSF13B, IFIT3, MX1, EPSTI1, PARP14, HERC5, and EIF2AK2 (Figure 4G). Functional enrichment analysis revealed that these upregulated genes are primarily involved in key pathways, such as interferon‐γ response (*p* < 0.001), mitochondrial oxidative phosphorylation (*p* = 0.016), neutrophil degranulation (*p* = 0.001), and leukocyte chemotaxis (*p* = 0.007) (Figure 4H–K, Figure S5A–P, Supporting Information). Further transcription factor enrichment analysis indicated that the expression of these genes during the acute exacerbation phase of MG is closely associated with key transcription factors, including STAT1 (*p* < 0.001), STAT2 (*p* < 0.001), STAT3 (*p* < 0.001), and MAFF (*p* = 0.003) (Figure S6A–P, Supporting Information). These findings are consistent with previous studies highlighting the activation characteristics of neutrophils during acute exacerbations, further supporting the critical role of neutrophils in the pathogenesis of MG disease progression.

### 2.5 Neutrophil Secretion of BAFF as a Key Factor in MG Acute Exacerbation

**Figure 5:**
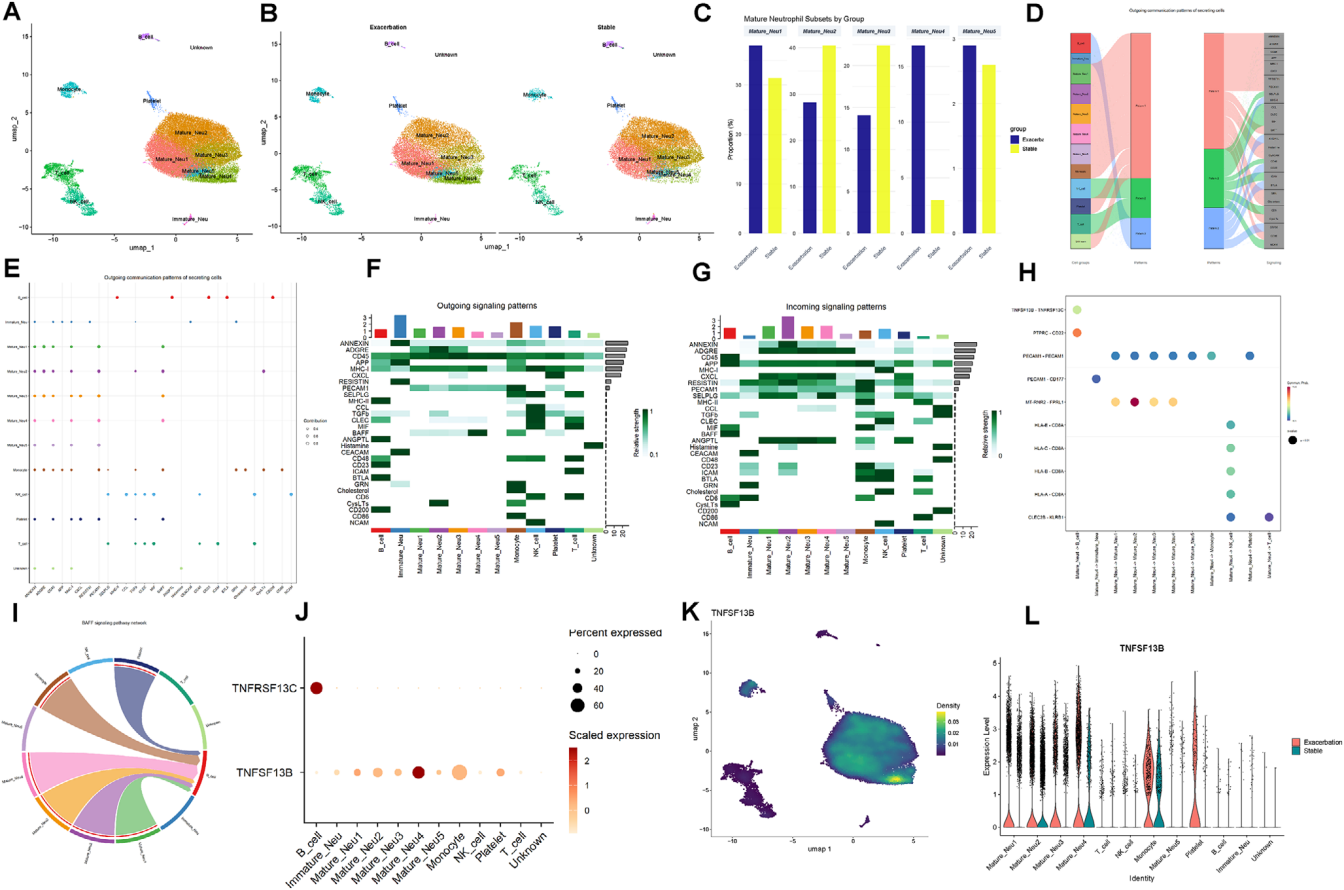
Intercellular communication analysis between the neutrophil subsets and B cells. A,B) UMAP representation of integrated peripheral blood cells, with mature neutrophils further subdivided into five distinct subpopulations (Mature Neu1‐5). C) The proportion of mature neutrophil subsets in the MG exacerbation and stable groups. D,E) Visualization of ligand‐receptor interactions and outgoing signaling from secreting cells in peripheral blood using Sankey D) and bubble plots E). F,G) Heatmaps showing outgoing F) and incoming G) signaling patterns in the two groups, with the color intensity of each tile representing the relative strength of signal output or input for each cell lineage and each L–R pair. H) Bubble plots demonstrate mature neutrophil subset 4 (Mature Neu4) ligand–receptor interactions with other peripheral blood cells. I) A chord diagram illustrates cell–cell communication between Mature Neu1, Mature Neu2, Mature Neu3, Mature Neu4, Mature Neu5, and B cells. J) Visualization of the BAFF signaling pathway network from mature neutrophils and other cell types to the remaining immune cells. K,L) Density plot K) and violin plot L) illustrating the expression levels of TNFSF13B in peripheral blood cells. Abbreviations: MG, myasthenia gravis; BAFF, B‐cell activating factor; TNFSF13B, tumor necrosis factor superfamily member 13B.

In recent years, neutrophils, as a critical component of the innate immune system, have been increasingly recognized for their involvement in autoimmune diseases,^[^ ^17^, ^18^, ^19^ ^]^ particularly through interactions with T and B cells.^[^ ^20^, ^21^, ^22^, ^23^ ^]^ In this study, we performed unsupervised clustering analysis to classify neutrophils into five subpopulations (**Figure** **5**A,B). In patients with MG, the proportion of mature neutrophil subpopulation 4 was significantly higher during acute exacerbation than during the stable phase (16.9% vs. 3.3%; *p* = 0.039; Figure 5C; Figure S7A,B, Supporting Information). In addition, the proportions of mature neutrophil subpopulations 2 (27.1% vs. 37.3%) and 3 (14.1% vs. 23.5%) were lower during acute exacerbation compared with the stable phase, although the differences did not reach statistical significance (*p* < 0.05; Figure 5C; Figure S7A,B, Supporting Information). Furthermore, mature neutrophil subpopulation 4 exhibited significant differences in differential gene expression and functional characteristics compared to the other four neutrophil subpopulations (Figure S7C–V, Supporting Information). To further explore the intercellular interactions, we used CellChat to analyze the ligand–receptor interactions between neutrophil subpopulations and other blood cell types in both groups (Figure S8A–O). The results revealed that the signaling patterns across all neutrophil subpopulations were consistent and primarily followed the pattern 1 pathway, including BAFF, CXCL, and MCH I(Figure 5D). Notably, in the outgoing signal analysis from CellChat, mature neutrophil subpopulation 4 exhibited prominent BAFF secretion signals (Figure 5E,F). In contrast, in the incoming signal analysis, BAFF was found to be the most significant receiving signal for B cells (communication probability = 0.055, *p* < 0.001, Figure 5G,H).

BAFF, also known as tumor necrosis factor ligand superfamily member 13B (TNFSF13B), is a key factor that promotes B cell survival, differentiation into plasma cells, and the maintenance of plasma cell longevity. MG, as a B‐cell‐mediated autoimmune disease, has a pathogenesis closely linked to plasma cell function. Previous studies have shown that neutrophils influence autoimmune‐reactive plasma cells through the secretion of BAFF.^[^ ^23^, ^24^, ^25^ ^]^ Based on this evidence, neutrophils may play an important role in the pathogenesis of MG. We further analyzed the information flow between BAFF and its receptor, tumor necrosis factor receptor superfamily member 13C, across different blood cell subpopulations. Our results showed that mature neutrophil subpopulation 4 was the main source of BAFF signaling (Figure 5I,J). Additionally, we observed that during the acute exacerbation of MG, the expression of BAFF in mature neutrophils was significantly higher compared to stable‐phase MG patients, with mature neutrophil subpopulation 4 showing the highest BAFF expression in the acute phase (Figure 5K,L). These results suggest that the high secretion of BAFF by increased neutrophil during the acute phase of MG may contribute to the immunopathology of acute exacerbation.

### 2.6 Bone Marrow Release of Neutrophils to Peripheral Blood as a Key Pathogenic Event in EAMG Induction

**Figure 6:**
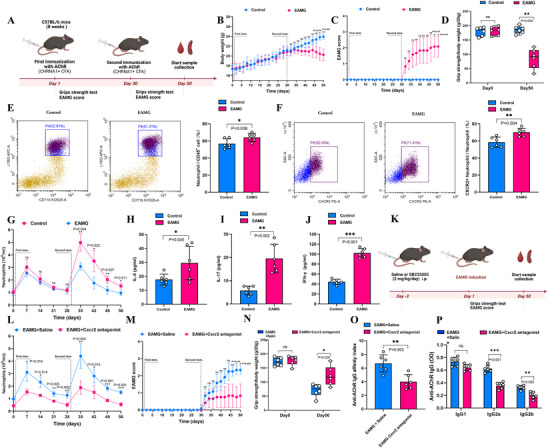
Bone marrow release of neutrophils to peripheral blood as a key pathogenic event in EAMG model induction. A) Schematic diagram of the experimental design for the EAMG model. B–D) After induction, EAMG mice showed significant changes in EAMG scores B), body weight C), and four‐limb grip strength at day 50 relative to baseline D). *n* = 6 per group. E,F) Flow cytometry analysis of bone marrow cells stained for CD45, CD11b, Ly6G, and CXCR2 revealed significantly higher proportions of total neutrophils (CD11b^+^ Ly6G^+^) E) and mature neutrophils (CD11b^+^ Ly6G^+^ CXCR2^+^) F) in EAMG mice compared to controls at day 50 post‐induction. *n* = 6 per group. G) Dynamic changes in peripheral blood neutrophil counts following EAMG induction. *n* = 6 per group. H–J) Significant increases in serum IL‐6 (H), TNF‐α (I), and IFN‐γ (J) levels in peripheral blood at day 50 relative to baseline. *n *= 6 per group. K) Schematic diagram of the experimental design for CXCR2 inhibitor treatment. L) Dynamic comparison of peripheral blood neutrophil counts between CXCR2 inhibitor‐treated and control groups after EAMG induction. *n* = 6 per group. M,N) Changes in EAMG scores M) and four‐limb grip strength relative to baseline at day 50 N) in the CXCR2 inhibitor‐treated group. *n* = 6 per group. O) At day 50, anti‐AChR IgG affinity index was measured by ELISA, calculated as the OD at 450 nm minus the OD at 630 nm. *n* = 6 per group. P) At day 50, anti‐AChR IgG1, IgG2a, and IgG2b isotype titers were quantified by ELISA, expressed as OD at 450 nm minus OD at 630 nm. *n* = 6 per group. Abbreviations: EAMG, experimental autoimmune myasthenia gravis; AChR, acetylcholine receptor; OD, optical density; ELISA, enzyme‐linked immunosorbent assay. Data are presented as mean ± 95% CI. ns, not significant; *p < 0.05; **p < 0.01; ***p < 0.001. *p*‐values were calculated using two‐way ANOVA with Sidak's post hoc analysis B,C,G,L,M,P); Mann–Whitney U test with Sidak correction for multiple comparisons D,N); or unpaired *t*‐test E,F,H,I,J,O). In all cases, *n* indicates the number of samples per group.

To further investigate whether the increase in neutrophils is a result of MG disease progression or a contributing factor to the acute exacerbation of MG, we established an EAMG mouse model (**Figure** **6**A). During the model induction, 83.3% of the mice successfully developed EAMG, exhibiting progressive muscle weakness and significantly increased EAMG scores, indicating successful simulation of the MG acute exacerbation phase (Figure 6B‐D). After successful modeling, we extracted bone marrow from the mice and performed flow cytometry analysis, which revealed a significant increase in the proportion of neutrophils (64.1% versus 56.7%, *p* = 0.038) and mature neutrophils (70.1% vs. 58.5%, *p* = 0.004) in the bone marrow of EAMG mice compared with control mice (Figure 6E,F). Peripheral blood analysis revealed that both the neutrophil count (0.66 ± 0.05×10^6^ mL^−1^ vs. 0.42±0.04×10^6^ mL^−1^, *p* < 0.001) and ratio (16.0% vs. 9.9%, *p* < 0.001) were elevated in EAMG mice at 50 days after model induction (Figure S9A,B, Supporting Information). Additionally, a more detailed time‐course analysis showed that neutrophil counts significantly increased following each of the two adjuvant and antigen administrations, displaying a characteristic bimodal pattern (Figure 6G). Moreover, we observed significantly elevated levels of cytokines including IFN‐γ (102.2 ± 8.3 pg mL^−1^ vs. 44.4 ± 4.7 pg mL^−1^, *p* < 0.001), IL‐6 (29.5 ± 10.9 pg mL^−1^ vs. 17.7 ± 3.6 pg mL^−1^, *p* = 0.045), IL‐17 (19.5 ± 5.6 pg mL^−1^ vs. 5.8 ± 1.7 pg mL^−1^, *p* = 0.002), and TNF‐α (60.5 ± 11.4 pg mL^−1^ vs. 30.4 ± 5.0 pg mL^−1^, *p* = 0.001) in the peripheral blood of the EAMG group compared with the control group (Figure 6H–J; Figure S9C, Supporting Information). These findings are consistent with the increased neutrophils and inflammatory state observed in the bone marrow and peripheral blood of MG acute exacerbation patients, further supporting the critical role of neutrophils in the immune response during the acute exacerbation of MG.

To investigate the role of neutrophils during the acute exacerbation of MG, we first established an EAMG model and intervened with the CXCR2 inhibitor SB225002 (2 mg kg^−1^ day^−1^, intraperitoneal injection) to explore the mechanism of neutrophil release from the bone marrow into the peripheral blood (Figure 6K). The results showed that, compared to the control group, the SB225002‐treated group exhibited a significant reduction in peripheral blood neutrophil counts throughout the experimental period, along with markedly lower EAMG scores and improved four‐limb grip strength (Figure 6L–N). Additionally, humoral immune analysis revealed that the SB225002 group had significantly decreased anti‐AChR IgG affinity (*p* = 0.003), IgG2a (*p* < 0.001), and IgG2b (*p* = 0.002) subtype levels compared to the control group, while no significant difference was observed in IgG1 levels (Figure 6O,P). To further clarify the specific role of neutrophils, we selectively depleted neutrophils using the anti‐Ly6G monoclonal antibody (200 µg per mouse, administered every 3 days) and then established the EAMG model (Figure S10A, Supporting Information). The results showed that the EAMG scores and four‐limb grip strength levels of the neutrophil‐depleted group were highly consistent with those of the SB225002 intervention group (Figures S10B–D, Supporting Information). Meanwhile, anti‐AChR IgG affinity (*p* < 0.001), IgG2a (*p* < 0.001), and IgG2b (*p* = 0.024) subtype levels were also significantly downregulated in the neutrophil‐depleted group, while no significant differences in IgG1 levels were observed (Figure S10E,F, Supporting Information).

In EAMG mice, immunohistochemical analysis of spleen tissue revealed a significant increase in neutrophil infiltration within the red pulp compared to controls (Neutrophils/total nucleated cells in splenic red pulp: 10.7% vs. 6.3%, *p* < 0.001) (Figures S11A,B, Supporting Information). Subsequent quantitative PCR analysis demonstrated that EAMG induction significantly elevated the transcription levels of neutrophil adhesion molecules, matrix‐degrading enzymes, and pro‐inflammatory cytokines involved in neutrophil chemotaxis (Figures S11C–E, Supporting Information). These findings suggest that the EAMG model triggers robust neutrophil mobilization from the bone marrow to peripheral blood and immune organs like the spleen, potentially playing a critical role in the disease pathology.

### 2.7 Regulation of BAFF Secretion by Neutrophils Through IFN‐γ Plays a Key Role in the Induction of the EAMG Model

**Figure 7:**
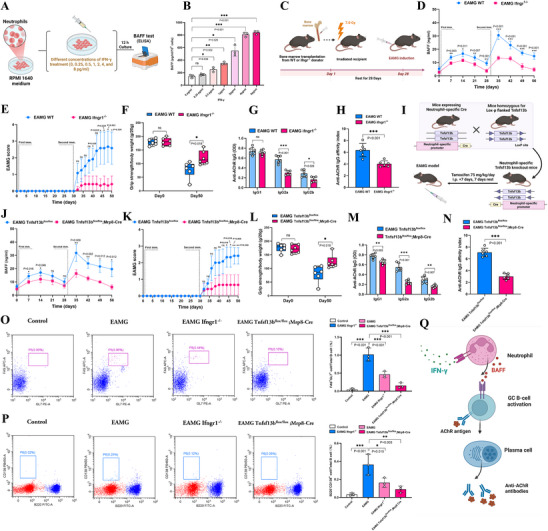
IFN‐γ promotes BAFF production by neutrophils and enhances anti‐AChR antibody responses via B cell activation in EAMG. A) Schematic diagram of the in vitro culture experiment using human neutrophils. B) BAFF levels in the culture supernatant of human neutrophils increased significantly in a dose‐dependent manner upon IFN‐γ stimulation; *n* = 3 per group. C) Experimental design for the establishment of EAMG in bone marrow chimeric mice lacking the Ifngr1 gene. D) Dynamic changes in serum BAFF levels in Ifngr1^−/−^ bone marrow chimeric EAMG mice after disease induction. *n* = 6 per group. E,F) Ifngr1^−/−^ bone marrow chimeric EAMG mice showed significantly reduced EAMG scores E) and improved four‐limb grip strength at day 50 relative to baseline F) compared to controls. *n* = 6 per group. G,H) At day 50 post‐induction, Ifngr1^−/−^ bone marrow chimeric mice exhibited significantly lower serum levels of anti‐AChR IgG2a and IgG2b antibodies G) and reduced antibody affinity indices H). *n* = 6 per group. I) Schematic of EAMG induction in neutrophil‐specific Tnfsf13b CKO mice. J) Dynamic changes of serum BAFF concentrations in neutrophil‐specific Baff knockout mice. *n* = 6 per group. K,L) Neutrophil‐specific Tnfsf13b CKO EAMG mice showed significantly lower EAMG scores K) and improved four‐limb grip strength relative to baseline at day 50 L). *n* = 6 per group. M,N) At day 50 post‐induction, these mice exhibited significantly decreased levels of anti‐AChR IgG1, IgG2a, and IgG2b antibodies M) and lower antibody affinity indices N). *n *= 6 per group. O,P) Flow cytometric analysis of splenic B220, CD138, Fas, and GL7 expression at day 50 post‐induction showed significantly reduced proportions of germinal center B cells (Fas⁺ GL7⁺) O) and plasma cells (B220^−^ CD138⁺) P) in Ifngr1^−/−^ bone marrow chimeric and neutrophil‐specific Tnfsf13b knockout mice compared to wild‐type EAMG mice. *n* = 6 per group. Q) Proposed model: IFN‐γ promotes BAFF expression in neutrophils, which in turn enhances B cell proliferation and differentiation into plasma cells that produce anti‐AChR antibodies. Abbreviations: EAMG, experimental autoimmune myasthenia gravis; BAFF, B‐cell activating factor; Tnfsf13b, tumor necrosis factor superfamily member 13B; CKO, conditional knockout; AChR, acetylcholine receptor; SEM, standard error of the mean. Data are presented as mean ± 95% CI. ns, not significant; *p < 0.05; **p < 0.01; ***p < 0.001. *p*‐values were calculated using one‐way ANOVA with Dunnett's multiple comparisons test B); one‐way ANOVA with Tukey's multiple comparisons test O,P); two‐way ANOVA with Sidak's post hoc analysis D,E,J,K); Mann–Whitney U test with Sidak correction for multiple comparisons F,G,L,M); or unpaired *t*‐test H,N). In all cases, *n* refers to the number of samples per group.

Our previous results suggest a positive correlation between the level of IFN‐γ and clinical severity in MG patients (Figure S4C, Supporting Information). Notably, the increase in BAFF secretion by neutrophils is influenced by multiple factors, and IFN‐γ has been suggested as a potential upstream signaling molecule capable of inducing BAFF secretion.^[^ ^26^, ^27^ ^]^ Moreover, our GSEA results revealed that the IFN‐γ response pathway was activated in blood neutrophils during acute MG exacerbation (Figure 5H, Supporting Information). To determine whether IFN‐γ is a key factor in stimulating neutrophils to secrete BAFF, and considering that the serum IFN‐γ levels in the majority of MG and HC subjects fall within the range of 0–8 pg mL^−1^, we stimulated neutrophils in vitro using IFN‐γ concentrations within this range. The results showed a dose‐dependent increase in BAFF secretion by neutrophils as the IFN‐γ concentration increased. Notably, at 4 pg mL^−1^ (808.1 ± 30.2 pg mL^−1^ per 10^7^ cells vs. 140.5 ± 21.1 pg mL^−1^ per 10^7^ cells, *p* < 0.001) and 8 pg mL^−1^ (840.2 ± 24.4 pg mL^−1^ per 10^7^ cells vs. 140.5 ± 21.1 pg mL^−1^ per 10^7^ cells, *p* < 0.001), the differences compared to the 0 pg mL^−1^ group were particularly significant. (**Figure** **7**A,B). Taken together, we hypothesize that IFN‐γ activates neutrophils, driving abnormal BAFF secretion in vivo.

To elucidate the mechanism by which IFN‐γ signaling regulates the pathological progression of MG through BAFF secretion from neutrophils, we first established Ifngr1^−/−^ bone marrow chimeric models by transplanting Ifngr1^−/−^ donor bone marrow into wild‐type recipients (Figure 7C). After EAMG induction, Ifngr1^−/−^ chimeric mice showed a significant reduction in serum BAFF levels compared to control mice (Figure 7D), along with decreased EAMG scores and improved four‐limb grip strength (Figure 7E,F), and significantly reduced anti‐AChR IgG levels and affinity (Figure 7G,H), with all *p*‐values < 0.05. To further validate this, we used neutrophil‐specific BAFF CKO mice (Mrp8Cre‐ERT2^+/−^‐Tnfsf13b^fl/fl^) to induce EAMG (Figure 7I). The results showed that, compared to controls (Mrp8Cre‐ERT2^−/−^‐Tnfsf13b^fl/fl^), neutrophil‐specific BAFF CKO mice had a significant decrease in serum BAFF levels (Figure 7J), along with reduced EAMG scores and improved four‐limb grip strength (Figure 7K,L), and a significant reduction in anti‐AChR IgG levels and antibody affinity (Figure 7M,N) after EAMG induction, all of which reached statistical significance at *p* < 0.05. Flow cytometric analysis further confirmed that EAMG mice exhibited a significant increase in the proportion of splenic germinal center (GC) B cells (1.01% vs. 0.04%, *p* < 0.001) and plasma cells (0.36% vs. 0.03%, *p* < 0.001) in the spleen compared with controls (Figure 7O,P). In contrast, both Ifngr1^−/−^ chimeric mice and neutrophil‐specific BAFF CKO mice exhibited markedly reduced proportions of GC B cells (0.46% in the EAMG group vs. 0.15% in Ifngr1^−/−^ chimeric mice and 0.10% in BAFF CKO mice) and plasma cells (0.36% in the EAMG group vs. 0.16% in Ifngr1^−/−^ chimeric mice and 0.09% in BAFF CKO mice) (Figure 7O,P), and these reductions were statistically significant compared with the EAMG group (all *p* < 0.05). To further establish a causal relationship between BAFF and MG pathogenesis, we employed an EAMG model treated with an anti‐BAFF monoclonal antibody (2 mg kg^−1^ day^−1^, intraperitoneally, twice weekly; Figure S12C, Supporting Information). Anti‐BAFF mAb administration resulted in a sustained reduction of peripheral BAFF levels compared with the control IgG group (Figure S12D, Supporting Information), which was accompanied by significantly lower EAMG clinical scores and improved four‐limb grip strength throughout the experimental period (Figure S12E,F, Supporting Information). Humoral immune analysis showed that anti‐BAFF mAb treatment markedly reduced anti‐AChR IgG affinity (*p* < 0.001) and IgG2a (*p* = 0.001) and IgG2b (*p* = 0.030) subclass levels, without affecting IgG1 (Figure S12G,H, Supporting Information). Flow cytometric analysis further demonstrated a significant decrease in the proportion of splenic GC B cells (0.15% vs. 1.00%, *p* = 0.002) and plasma cells (0.03% vs. 0.25%, *p* = 0.003) in the anti‐BAFF mAb group compared with controls (Figure S12I,J, Supporting Information). Taken together, our findings suggest that the IFN‐γ signaling pathway drives neutrophil secretion of endogenous BAFF, which in turn activates the differentiation of splenic GC B cells into autoantibody‐producing plasma cells (Figure 7Q, Supporting Information), supporting the presence of an immunoregulatory axis during MG acute exacerbation.

### 2.8 Exploratory Analysis of Telitacicept Efficacy in MG Patients Stratified by Baseline Neutrophil Level

**Figure 8:**
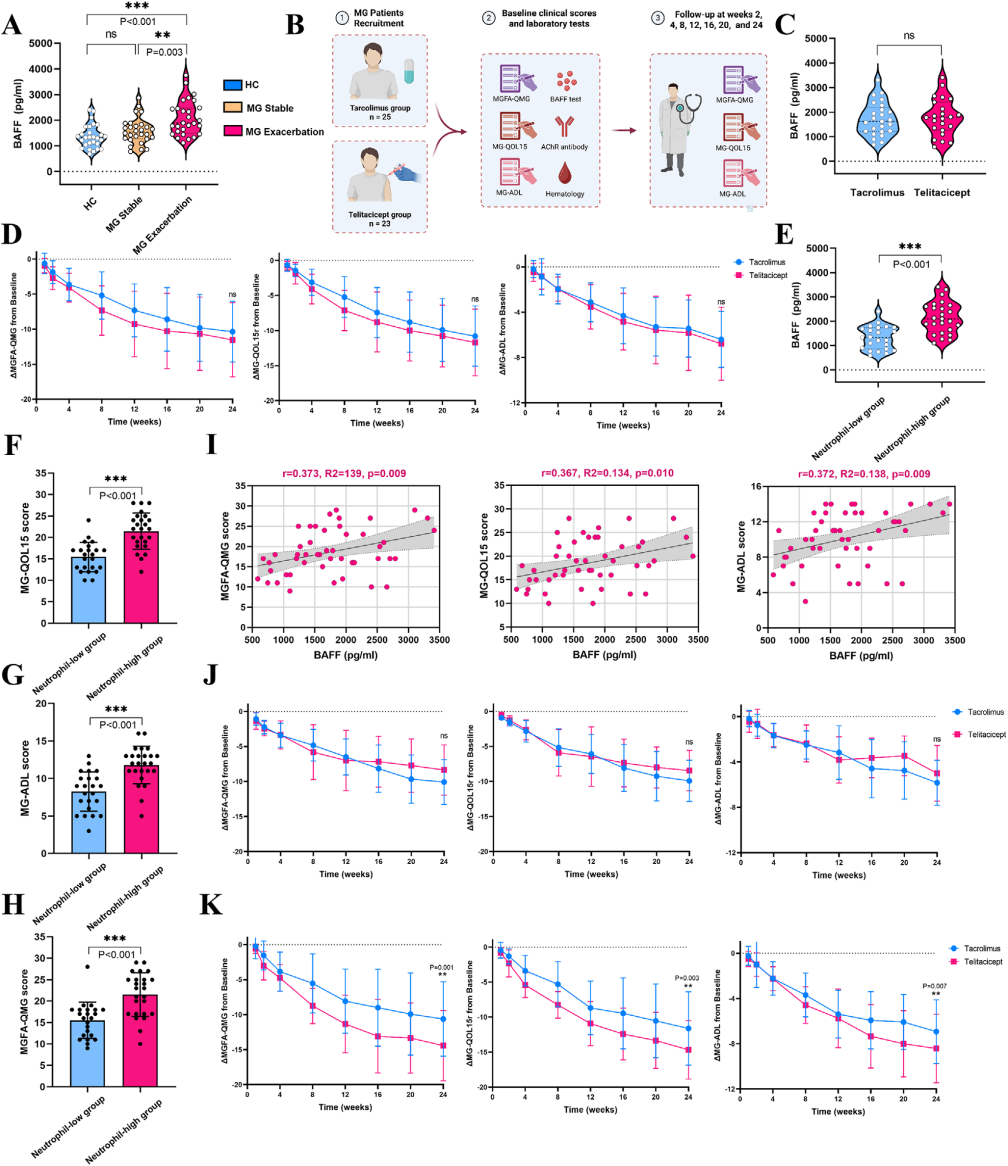
Clinical effects of telitacicept versus tacrolimus in the treatment of MG and post‐hoc analyses. A) Serum BAFF levels in healthy controls (*n* = 28), MG stable phase (*n* = 28), and MG exacerbation phase (*n* = 28). B) Schematic overview of the clinical trial design for telitacicept versus tacrolimus treatment in MG. C) Baseline serum BAFF by treatment arm (tacrolimus, *n *= 25; telitacicept, *n *= 23). D) Comparison of the 24‐week change from baseline in MGFA‐QMG (left), MG‐QOL15r (middle), and MG‐ADL (right) by treatment group (tacrolimus, *n *= 25; telitacicept, *n* = 23). E) Baseline serum BAFF in the low‐neutrophil (*n* = 23) and high‐neutrophil (*n *= 25) subgroups. F–H) Baseline MGFA‐QMG (F), MG‐QOL15r G), and MG‐ADL H) by neutrophil‐based subgroup. I) Spearman correlations between baseline serum BAFF and MGFA‐QMG (left), MG‐QOL15r (middle), and MG‐ADL (right) in patients with MG (*n* = 48). J–K) Within‐subgroup comparisons of 24‐week change in MGFA‐QMG (left), MG‐QOL15r (middle), and MG‐ADL (right) between tacrolimus and telitacicept in the low‐neutrophil subgroup (*n* = 23) J) and the high‐neutrophil subgroup (*n* = 25) K). Abbreviations: MG, myasthenia gravis; BAFF, B‐cell activating factor; MGFA‐QMG, Myasthenia Gravis Foundation of America Quantitative Myasthenia Gravis; MG‐QOL15r, Myasthenia Gravis Quality of Life 15‐item revised; MG‐ADL, Myasthenia Gravis Activities of Daily Living. Data are presented as mean ± 95% CI. ns, not significant; *p < 0.05; **p < 0.01; ***p < 0.001. *p*‐values were calculated using one‐way ANOVA with Tukey's multiple comparisons test A); unpaired *t*‐test C,E,F,G,H); Spearman correlation analysis I); ANCOVA with adjustments for baseline clinical scores D); or ANCOVA with adjustments for baseline clinical scores, neutrophil‐based subgroup, and the treatment and neutrophil‐based subgroup interaction J,K). In all instances, *n* refers to the number in each group.

Given the potential key role of elevated BAFF levels in the pathogenesis of acute exacerbation in MG, we performed enzyme‐linked immunosorbent assay (Elisa) quantification of BAFF in clinical serum samples. Serum BAFF levels were higher in the acute exacerbation group (2048.9 ± 628.9 pg mL^−1^) than in the stable phase group (1696.0 ± 540.3 pg mL^−1^, *p* = 0.003) and healthy controls (HCs) (1391.7 ± 464.6 pg mL^−1^, *p* < 0.001), with no significant difference between the stable phase group and HCs (*p* > 0.05) (**Figure** **8**A). This result suggests that BAFF may be an important therapeutic target in acute MG exacerbations. Currently, telitacicept, a biologic agent approved by the National Medical Products Administration of China,^[^ ^28^ ^]^ targets both BAFF and APRIL and has demonstrated significant efficacy in improving clinical scores in MG patients in a phase II clinical trial.^[^ ^29^ ^]^ Based on these findings, we conducted a prospective clinical study at Peking University People's Hospital, enrolling 48 MG patients, who were divided into a telitacicept therapy group (*n *= 23) and a tacrolimus group (*n *= 25) (Figure 8B).

At baseline, there were no significant differences between the tacrolimus and telitacicept groups in terms of gender, age, baseline MG clinical scores, or baseline BAFF levels (Figure 8C; Table S4, Supporting Information). From baseline to week 24, there were no between‐group differences in the change in Myasthenia Gravis Foundation of America‐Quantitative Myasthenia Gravis (MGFA‐QMG), Myasthenia Gravis Quality of Life 15‐Item Revised (MG‐QOL15r), and Myasthenia Gravis Activities of Daily Living (MG‐ADL) scales (all *p* > 0.05) (Figure 8D; Table S5, Supporting Information). When stratified by baseline absolute neutrophil count (ANC; cohort median 4.5 × 10⁹ L^−1^), the high‐neutrophil subgroup had higher baseline BAFF levels and more severe clinical scores than the low‐neutrophil subgroup (all *p* < 0.05, Figure 8E–H; Table S6, Supporting Information). Besides, baseline serum BAFF levels were positively correlated with MG clinical scores (Figure 8I). Given this heterogeneity, we performed a post‐hoc analysis by adding the treatment × ANC‐based subgroup interaction to the baseline‐adjusted ANCOVA for each endpoint and estimating within‐stratum contrasts. The interaction between treatment and ANC‐based subgroup was significant for MGFA‐QMG (*p* = 0.003), MG‐QOL15r (*p* = 0.008), and MG‐ADL (*p* = 0.014). In the high‐neutrophil subgroup, outcomes favored telitacicept over tacrolimus, with adjusted mean (LSM) differences (tacrolimus–telitacicept) of 3.8 (95% confidence interval (CI), 1.7–5.9; *p* = 0.001) for MGFA‐QMG, 3.4 (95% CI, 1.4–5.9; *p* = 0.003) for MG‐QOL15r, and 1.9 (95% CI, 0.7–3.1; *p* = 0.007) for MG‐ADL. No between‐treatment differences were observed in the low‐neutrophil subgroup (Figure 8J,K; Table S7, Supporting Information).

The power analysis based on the MGFA‐QMG score showed that Cohen's *f* ^2^ for the treatment × ANC‐based subgroup interaction was 0.28, yielding an estimated post‐hoc power of 0.93 (α = 0.05) to detect the observed interaction. Sensitivity analyses that further included the ANC‐based subgroup × baseline score interaction to account for potential confounding by baseline severity confirmed the robustness of these findings, with results continuing to favor telitacicept in the high‐neutrophil subgroup (Table S8, Supporting Information). Based on our previous mechanistic studies, we hypothesize that in MG patients with a high neutrophil state, neutrophil chemotaxis and BAFF secretion in the bone marrow and peripheral blood may exacerbate MG severity. This mechanism may explain the greater targeted efficacy of telitacicept observed in the high neutrophil subgroup. Therefore, our findings provide new evidence for individualized precision immunotherapy strategies. We recommend that future phase III clinical trials include baseline neutrophil count as an important stratification factor and further investigate its molecular mechanisms in myeloid‐lymphoid immune crosstalk.

## 3 Discussion

**Figure 9:**
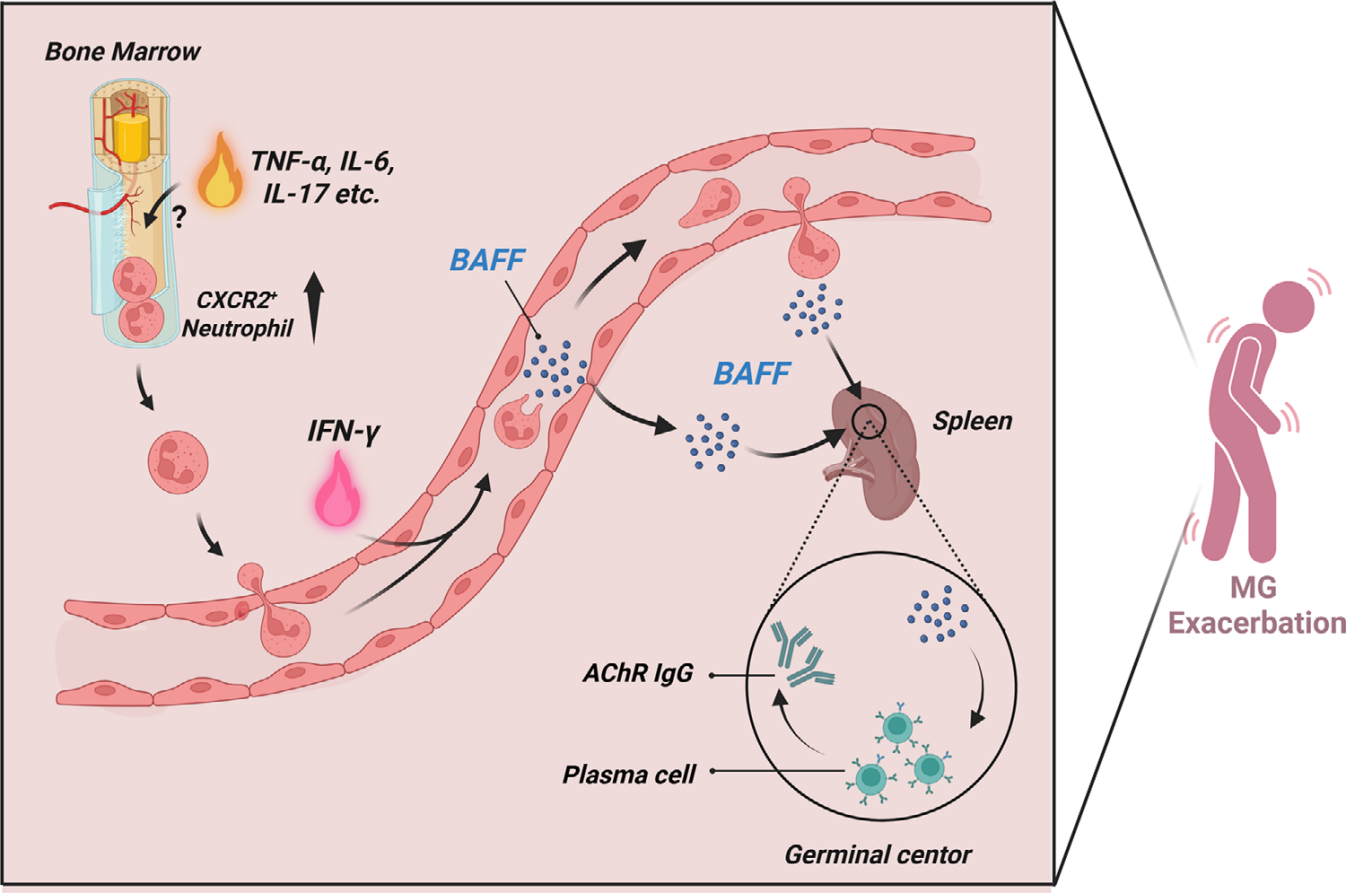
Hypothetical mechanism model of this study. During MG exacerbation, the bone marrow exhibits enhanced myeloid differentiation, leading to increased production and mobilization of neutrophils into the peripheral blood. Under the stimulation of IFN‐γ, these circulating neutrophils secrete excessive levels of BAFF. Elevated BAFF levels promote the differentiation of germinal center B cells into antibody‐secreting plasma cells in secondary lymphoid organs such as the spleen. This process results in increased production of anti‐AChR antibodies, thereby exacerbating MG pathology. Abbreviations: MG, myasthenia gravis; BAFF, B‐cell activating factor; AChR, acetylcholine receptor.

This study systematically elucidates the immune dynamics during the acute exacerbation phase of MG, with a particular focus on the previously unresolved immune regulatory mechanisms underlying disease pathogenesis. By integrating single‐cell transcriptomics and clinical sample analysis, we provide the first comprehensive characterization of the cellular composition and molecular features of the bone marrow and peripheral immune system during acute MG exacerbation. The key findings include: 1) During acute exacerbation, the proportion of mature neutrophils in the bone marrow increases significantly, exhibiting functional phenotypes related to inflammatory responses and immune cell migration; 2) a concurrent increase in peripheral blood neutrophils that contribute to a unique immune microenvironment through the secretion of BAFF; 3) mechanistic studies based on the EAMG model suggest that neutrophil‐derived BAFF may enhance plasma cell responses, thereby contributing to disease progression during exacerbation; and (4) supporting evidence from a prospective clinical trial showing that BAFF‐targeted therapy exhibits superior clinical efficacy in MG patients with high neutrophil counts (**Figure** **9**). Collectively, these findings support an important role of the neutrophil‐BAFF‐plasma cell axis in acute MG exacerbation.

Bone marrow, as an important immune organ, not only serves as the primary site for hematopoiesis but also plays a crucial role in maintaining immune homeostasis, especially during infectious diseases, where the proliferation of myeloid cells aids in pathogen clearance.^[^ ^30^, ^31^, ^32^ ^]^ In this study, we found through scRNA‐seq and flow cytometry analysis that the bone marrow acts as an early responder during the acute exacerbation of MG, capable of sensing immune system activation and promoting the differentiation of the neutrophil lineage into mature neutrophils. Additionally, the elevated levels of cytokines such as IFN‐γ and IL‐17 in the peripheral blood of MG patients during acute exacerbation may serve as key factors stimulating neutrophil differentiation in the bone marrow. Neutrophils, the most abundant white blood cells in the immune system, typically play a role in the body's initial defense against infections, with strong phagocytic and antimicrobial functions.^[^ ^30^, ^33^ ^]^ However, recent studies have shown that neutrophils may also contribute to the development and progression of autoimmune diseases.^[^ ^18^ ^]^ Consistent with previous findings,^[^ ^17^, ^18^, ^19^ ^]^ we further confirmed that the proportion of neutrophils in the peripheral blood is positively correlated with the severity of MG, suggesting their deleterious pathogenic role. To validate this hypothesis, we employed the EAMG model. We found that both neutrophil depletion and inhibition of neutrophil migration from bone marrow to peripheral blood significantly suppressed EAMG severity. These results further support the pathogenic role of neutrophils in MG progression.

To further explore the relationship between neutrophils and other immune cells, we conducted an intercellular communication analysis. The results showed that during the MG exacerbation phase, BAFF signaling from mature neutrophil subset 4 significantly enhances communication with B cells, and the expression of BAFF was notably increased in this subset. BAFF, also known as TNFSF13B, is a member of the TNF ligand superfamily and plays a critical role in the maturation and survival of B cells.^[^ ^25^, ^34^ ^]^ Previous studies have demonstrated that elevated BAFF levels contribute significantly to the progression of autoimmune diseases such as systemic lupus erythematosus, Sjögren's syndrome, and idiopathic thrombocytopenic purpura.^[^ ^23^, ^35^, ^36^ ^]^ The increased secretion of BAFF by neutrophils is regulated by multiple factors, among which IFN‐γ is recognized as an upstream signaling factor for BAFF induction.^[^ ^26^, ^27^ ^]^ Supporting this, our bioinformatics analysis demonstrated significant activation of the IFN‐γ response pathway in neutrophils from the peripheral blood of MG patients during acute exacerbation, consistent with elevated circulating IFN‐γ levels. The experimental findings indicate that IFN‐γ stimulation markedly upregulates BAFF secretion by human neutrophils, exhibiting a clear dose‐dependent pattern within the physiologically relevant concentration range. These findings indicate that IFN‐γ serves as a key upstream inducer of neutrophil‐derived BAFF in MG acute exacerbation. Moreover, experimental studies employing Ifngr1^−/−^ bone marrow chimeric mice and neutrophil‐specific BAFF knockout mice further confirmed the critical role of the IFN‐γ‐BAFF axis in mediating neutrophil involvement in the acute exacerbation of MG. Additionally, previous studies using EAMG animal models have also demonstrated that IFN‐γ knockout significantly suppresses disease onset and progression,^[^ ^37^ ^]^ although the precise molecular mechanisms underlying this effect remain to be fully elucidated. Collectively, these findings suggest that IFN‐γ contributes to BAFF secretion by neutrophils, and this axis is involved in the pathogenesis of MG exacerbation.

This study has significant clinical implications, suggesting that targeted intervention against neutrophil‐derived BAFF may serve as an important therapeutic strategy to inhibit MG disease progression. Previous phase II clinical trials showed that belimumab, a BAFF‐specific monoclonal antibody, did not significantly improve clinical scores in MG patients.^[^ ^38^ ^]^ In contrast, telitacicept, a dual‐target biologic inhibiting both BAFF and APRIL, demonstrated significant improvements in clinical scores and quality of life in MG patients in a recent phase II clinical trial conducted in China.^[^ ^29^ ^]^ Although both drugs target the key molecule BAFF involved in B cell survival, the results of these two studies differ to some extent. Considering that patients enrolled in the earlier study had significantly lower MGFA‐QMG scores, we speculate that they were mostly MG patients with relatively stable disease, which may be an important factor contributing to the negative outcome of that clinical trial. Given that telitacicept is the only BAFF‐targeting drug approved by the China National Medical Products Administration and successfully used in MG treatment, ^[^ ^30^, ^31^ ^]^ we conducted a clinical study using this drug as a therapeutic option for MG. However, in our study, the telitacicept group did not show significant improvement in overall clinical scores at 6 months compared to the tacrolimus group. This discrepancy with previous studies may be related to differences in patient characteristics. These include age distribution, disease severity, and the control group (tacrolimus treatment).

To further clarify this discrepancy, we performed a post hoc analysis based on baseline neutrophil levels. The results showed that telitacicept demonstrated significant clinical efficacy in the high‐neutrophil subgroup. This enhanced efficacy may be related to the higher baseline BAFF levels and more severe clinical scores observed in this subgroup. However, even within the low‐neutrophil subgroup, telitacicept did not show a clear therapeutic advantage over tacrolimus, suggesting that baseline neutrophil status may influence the effectiveness of BAFF‐targeted therapies. Future studies should consider incorporating baseline neutrophil levels as a potential predictive factor for treatment response. In summary, these findings provide a new perspective for evaluating the indications for BAFF‐targeted and related therapies in MG, with the goal of achieving personalized treatment and more precise control over MG progression.

This study has several limitations. First, although our main findings highlight the enhanced differentiation of bone marrow neutrophils and their migration to peripheral blood during acute exacerbation of MG, the precise mechanisms and driving factors underlying myeloid hematopoiesis remain incompletely understood. Addressing this gap may provide new therapeutic targets for MG and other autoimmune diseases during exacerbation phases. Second, there is currently a lack of animal models specifically targeting the acute exacerbation phase of MG. In this study, we used the EAMG mouse model as a substitute; however, this model exhibits differences in certain immune characteristics compared to human MG and may not fully recapitulate the pathological complexity of MG acute exacerbation. Therefore, future research should focus on developing more representative animal models of MG acute exacerbation to overcome these shortcomings. Third, the 24‐week follow‐up in this study, consistent with that of previous MG randomized controlled clinical trials, reflects only short‐term efficacy. However, the durability of this effect and the long‐term safety profile remain to be determined in future studies. Finally, attention should be paid to the diverse sources of BAFF in vivo, including neutrophils, monocytes, and dendritic cells.^[^ ^39^, ^40^ ^]^ Our study has not excluded the potential contributions of BAFF derived from non‐neutrophil sources to the pathogenesis of MG acute exacerbation. Subsequent work should further clarify the specific roles of BAFF from various immune cell types.

## 4 Conclusion

In summary, our study provides novel insights into the immune dynamics and regulatory mechanisms involved in the acute exacerbation of MG. We have shown that neutrophils, through the secretion of BAFF, contribute to the progression and exacerbation of MG. These findings reveal the importance of the neutrophil‐BAFF‐plasma cell axis in MG pathology and propose new therapeutic strategies targeting neutrophil functional regulation or blocking the BAFF signaling pathway. Moreover, our study highlights the significant clinical implications of neutrophil‐derived BAFF in MG treatment, emphasizing the potential for personalized therapeutic interventions.

## 5 Experimental Section

### Patients

From February 2024 to April 2025, a total of 142 participants, including 28 age‐ and sex‐matched HCs and 114 patients with MG, were recruited at Peking University People's Hospital. Participants were categorized into three cohorts: Cohort 1 included 3 patients with acute exacerbation of MG and 3 patients with stable MG, who provided peripheral blood and bone marrow samples for scRNA‐seq; Cohort 2 included 28 HCs, 30 patients with acute exacerbation of MG, and 30 patients with stable MG, aimed at evaluating the correlation between blood parameters, serum cytokines, and MG severity; Cohort 3 comprised 48 MG patients participating in a 24‐week prospective clinical trial (Trial registration: ChiCTR2400084715), comparing the clinical efficacy of two treatment regimens: tacrolimus (*n* = 25) versus telitacicept (*n* = 23), with the primary endpoint being improvment in MG clinical scores.

MG patients met the following inclusion criteria: positive for anti‐AChR antibodies, MGFA stage II–IVa, no prior use of corticosteroids or immunosuppressants, and no active cancers, infections, or other autoimmune diseases. Diagnosis was based on clinical presentation, neostigmine testing, nerve conduction studies, and anti‐AChR antibody testing. Baseline disease severity was assessed using MGFA‐QMG, MG‐ADL, and MG‐QOL15r scores. Acute exacerbation of MG is defined as an increase of ≥ 5 points in the MGFA‐QMG score within the two weeks prior to admission, with a total score of ≥ 8 points.^[^ ^7^ ^]^ The contribution of ocular symptoms to the MGFA‐QMG score should not exceed 5 points.^[^ ^7^ ^]^ Stable MG is defined as a fluctuation of no more than 3 points in the MGFA‐QMG score within the two weeks prior to admission. All participants provided written informed consent prior to study participation, and the study was approved by the Ethics Review Committee of Peking University People's Hospital (Approval No.2023PHB402‐006)

### scRNA‐seq Sample Preparation

For iliac bone marrow samples, the marrow was collected via posterior iliac crest puncture and lysed with ammonium‐chloride‐potassium (ACK) lysing buffer (Beyotime Biotechnology, China; Cat. C3702) at a 4:1 ratio on ice for 10 min, followed by 400 × g centrifugation and washing with 0.04% BSA/PBS. The cell concentration was adjusted to 700–1200 cells µL^−1^ after filtration through a 35 µm sieve. For peripheral blood, whole blood was mixed with ACK lysing buffer (1:9 ratio) for red blood cell lysis at room temperature for 5 min. Gradient centrifugation is used to remove debris. The suspension was filtered, and the cell concentration was adjusted to 500–1200 cells µL^−1^. Cell viability was confirmed to be over 90%, with less than 5% residual erythrocytes. Samples were processed within 1 hour of collection, kept at room temperature to prevent cold‐induced cell damage, and promptly loaded onto the 10x Genomics platform.

### Construction and Sequencing of scRNA‐seq Libraries

scRNA‐seq libraries were constructed using the Chromium Single Cell V(D)J Reagent Kit (10x Genomics) in accordance with the manufacturer's protocol. For each sample, approximately 1 × 10⁴ purified bone marrow mononuclear cells or peripheral blood mononuclear cells were encapsulated into Gel Beads‐in‐Emulsion. Sequencing was subsequently conducted on the Illumina NovaSeq 6000 platform.

### Quality Control and Filtering

Raw 10x Genomics data were processed using Cell Ranger (v6.1.2) with default parameters. Sequencing reads were aligned to the human reference genome GRCh38 V3.0.0 (including expanded mitochondrial and ribosomal gene annotations). Unique molecular identifiers and cell barcodes were mapped via the Cell Ranger pipeline. Subsequent analysis was performed in R software (version 4.3.3) using Seurat (version 4.3.0). The following quality control steps were implemented: 1) genes detected in ≥ 3 cells (min.cells = 3); 2) excluded cells with < 200 genes (min.features  = 200) or > 3500 genes; 3) removed cells with mitochondrial UMI content > 10% or ribosomal gene proportion >5%.

### Dimensionality Reduction and Visualization

Following quality control, highly variable genes were identified using the FindVariableFeatures function with variance‐stabilizing transformation. Transcriptomic data were log‐normalized (scale.factor = 10 000) and scaled to regress out mitochondrial gene effects. Batch effects were corrected via canonical correlation analysis‐based integration. Principal component analysis was performed on the integrated dataset, retaining 20 principal components based on cumulative variance contribution (> 80%) and elbow plot inflection points. UMAP was applied for nonlinear dimensionality reduction.

### Differential Gene Expression Analysis

Differentially expressed genes (DEGs) between a specific cell type and others were identified using the FindAllMarkers function (Seurat v5.0) with the Wilcoxon rank‐sum test. A log fold change threshold of logfc.threshold = 0.25 was applied, and genes were required to be expressed in at least 10% of the cells (min.pct = 0.1). For inter‐group comparisons, the FindMarkers function was used. Adjusted *p*‐values were calculated using Benjamini–Hochberg correction for false discovery rate (FDR) (FDR < 0.05), and DEGs were visualized using volcano plots, with genes labeled according to significance thresholds (e.g., |logFC| > 0.25 and FDR < 0.05).

### Enrichment Analyses

Gene Ontology (GO) and Kyoto Encyclopedia of Genes and Genomes (KEGG) pathway enrichment analyses were performed using the enrichGO and enrichKEGG functions (clusterProfiler v4.0, with Benjamini–Hochberg method for multiple testing correction, qvalueCutoff = 0.05). Additionally, to further investigate immune pathways and transcription factors, we selected Hallmark immune signature gene sets and transcription factor‐related gene sets for enrichment analysis. Upregulated DEGs were prioritized for biological interpretation. Significantly enriched pathways and terms were defined as those with FDR < 0.05 and |log2FoldChange| > 0.5.

### Assessment of Receptor/Ligand Interactions

Cell–cell interactions and dysregulated signaling pathways in peripheral blood cells were systematically evaluated using CellChat (v0.0.2). The analysis was centered on differentially expressed ligands and receptors in peripheral blood neutrophils and other immune cell populations. Neutrophil‐B cell interaction dynamics were further investigated based on reclustered neutrophil subsets. Standardized data were processed according to the official workflow: CellChat objects were constructed, and the “Secreted Signaling” pathway subset from the CellChatDB.human database was selected as the analytical framework. Putative ligand–receptor pairs were identified using default parameters, and interaction networks were visualized through circos plots to highlight pathway‐specific alterations.

### Pseudotime Trajectory Analysis

Pseudotime trajectory analysis was performed using Monocle2 (version 2.8.0) based on the DDR‐Tree algorithm under default settings. Marker genes obtained from Seurat clustering and quality‐controlled expression matrices were used as input. Cells were ordered using the orderCells function. Likelihood ratio tests were applied to identify pseudotime‐associated genes, followed by Benjamini–Hochberg FDR correction (FDR < 0.05), incorporating single‐cell RNA‐seq covariates such as batch effects. Branch‐specific dynamic changes were analyzed using BEAM to detect genes related to fate bifurcation.

### Cell Cycle Analysis

Cell cycle analysis was performed using the Seurat package. S and G2/M phase scores were calculated based on canonical gene sets, and each cell was assigned a cell cycle phase (G1, S, or G2/M) using the CellCycleScoring function. To minimize cell cycle‐related variation, S and G2/M scores were regressed during data scaling.

### Isolation and Culture of Neutrophils from Human Peripheral Blood

Neutrophils were isolated from human peripheral blood using a neutrophil isolation kit (Solarbio, China; Cat. P9040), following the manufacturer's protocol. Purified neutrophils were cultured in RPMI 1640 medium supplemented with 10% fetal bovine serum. Experimental conditions included treatment with recombinant human IFN‐γ (Biolegend, USA; Cat. 570206) stimulation at graded concentrations. Following incubation, BAFF levels in culture supernatants were quantified.

### Enzyme‐Linked Immunosorbent Assay

Serum samples from humans and mice were rapidly separated from whole blood by centrifugation at 1000 × *g* for 10 min and stored at −80 °C. Subsequently, ELISA kits were used according to the manufacturer's instructions to measure levels of BAFF, IL‐2, IL‐4, IL‐6, IL‐8, IL‐10, IL‐17, IFN‐γ, and TNF‐α in human serum, and BAFF, IL‐1β, IL‐4, IL‐6, IL‐10, IL‐17, IFN‐γ, and TNF‐α in mouse serum. The list of ELISA kits used is provided in Table S1 (Supporting Information).

### Mice

All animal experiments were approved by the Ethics Committee of Peking University People's Hospital. C57BL/6J mice were purchased from Beijing Vital River Laboratory Animal Technology Co., Ltd. Tnfrsf1a^−/‐^ mice, Mrp8Cre‐ERT2^+/−^ mice, and Tnfsf13b^fl/fl^ mice (5 weeks old, C57BL/6J background) were obtained from the Shanghai Model Organisms Center. Neutrophil‐specific conditional knockout (CKO) mice for Tnfsf13b were generated by crossing Mrp8Cre‐ERT2^+/−^ mice with Tnfsf13b^fl/fl^ mice, resulting in the Mrp8Cre‐ERT2^+/−^‐Tnfsf13b^fl/fl^ strain. To induce neutrophil‐specific gene deletion, tamoxifen (20 mg mL^−1^ in corn oil, Sigma–Aldrich, USA; Cat. T5648) was administered via intraperitoneal injection once daily for 7 consecutive days. Knockout efficiency was validated by isolating CD11b⁺ Ly6G⁺ neutrophils using flow cytometry, followed by PCR genotyping and Western blot analysis using antibodies against Tnfsf13b (Abcam, UK; Cat. ab8396). All animal procedures were reviewed and approved by the Institutional Animal Care and Use Committee of Chongqing Medical University (approval No. IACUC‐CQMU‐2024‐0726) and were conducted in accordance with relevant guidelines for animal protection, welfare, and ethics.

### EAMG Induction

C57BL/6J female mice (8 weeks old) were maintained under a 12‐h light/dark cycle with free access to food and water. To induce the EAMG model, the R97‐116 peptide (sequence DGDFAIVKFTKVLLDYTGHI, CSBio, USA) was emulsified with Complete Freund's Adjuvant (CFA, Sigma–Aldrich, USA; Cat. F5881) and supplemented with 1 mg of inactivated Mycobacterium tuberculosis H37RA (Difco Laboratories, USA; Cat. 231141) per mouse, following previously described protocols.^[^ ^41^, ^42^ ^]^ Under 3% isoflurane anesthesia, 100 µg of R97‐116 peptide was mixed 1:1 with CFA to form an emulsion and injected subcutaneously at the base of the tail (50 µL per side). On day 30, mice received a booster immunization with the same formulation. Body weight, four‐limb grip strength, and EAMG scores were assessed every other day. EAMG scores were defined as follows: grade 0, normal muscle strength with no weakness even after exercise (20–30 consecutive paw grips); grade 1, normal at rest but shows weakness after exercise, characterized by chin on the floor, inability to raise the head, hunched posture, and reduced mobility; grade 2, muscle weakness present at rest; and grade 3, moribund, dehydrated, and fully paralyzed; grade 4, death. Intermediate scores (e.g., 0.5, 1.5, 2.5, 3.5) were assigned to reflect partial symptoms between two adjacent grades. Four‐limb grip strength was measured using the grip strength meter (XR501, Shanghai Softron Biotechnology Co., Ltd.). The procedure was as follows: 1) Place the mouse's four limbs on the horizontal bar of the device and wait for a stable grip, then zero the instrument for baseline calibration; 2) The operator gently pulls the mouse's tail along its body axis at a constant speed of approximately 5 cm s^−1^, applying a traction force opposite to the grip direction; 3) When any limb releases the bar, the force sensor automatically records the peak grip strength, reflecting the maximal isometric contraction force of the mouse's limbs. Testing was conducted in a quiet environment, with no more than three measurements per day to avoid fatigue effects.

### Detection of Serum Anti‐R97‐116 Peptide IgG by ELISA

Serum anti‐R97‐116 peptide IgG antibodies were measured using ELISA. Microtiter plates (Corning Costar, USA; Cat. 3590) were coated with R97‐116 peptide (10 µg mL^−1^) at 4 °C overnight. The plates were then blocked with PBS containing 5% fetal bovine serum at 37 °C for 60 min. Serum samples diluted 1:100 in PBS were added to the wells and incubated at 37 °C for 2 h. After washing, biotinylated goat anti‐mouse IgG (ZSGB‐BIO, China; Cat. SAP‐9100) was added and incubated for 90 min. Streptavidin–horseradish peroxidase (HRP) conjugate (Invitrogen, Thermo Fisher Scientific, USA; Cat. SA10001) was then applied and incubated at 37 °C for 20 min. After further washing, color development was performed using tetramethylbenzidine (TMB, Sigma‐Aldrich, USA; Cat. T8665). Optical density was measured at 450 nm with a reference wavelength of 630 nm using a microplate reader. Each sample was tested in triplicate, and results were expressed as mean OD ± standard deviation.

### Determination of Relative Affinity of Serum Anti‐R97‐116 IgG

Microtiter plates (Corning Costar, USA; Cat. 3590) were coated with R97‐116 peptide (5 µg mL^−1^) and blocked with 5% fetal bovine serum. Diluted serum samples containing a defined level of anti‐R97‐116 antibodies were added and incubated. Subsequently, 200 µL of potassium thiocyanate (Sigma–Aldrich, USA; Cat. P3011) at varying concentrations was added in duplicate wells and incubated at room temperature for 15 min. This was followed by incubation with biotinylated goat anti‐mouse IgG (ZSGB‐BIO, China; Cat. SAP‐9100) and streptavidin‐HRP (Invitrogen, Thermo Fisher Scientific, USA; Cat. SA10001). After color development with TMB (Sigma–Aldrich, USA; Cat. T8665), OD was measured at 450 nm with 630 nm as a reference. The relative affinity was expressed as the affinity index, defined as the molarity of potassium thiocyanate required to reduce the OD by 50% compared to wells without potassium thiocyanate.

### Mice Routine Blood Tests

Mice's peripheral blood was collected via tail tip bleeding at multiple time points before and after EAMG induction to enable repeated, low‐volume sampling for hematological analysis. Complete blood count was performed using whole blood to measure hemoglobin, hematocrit, red blood cell, and white blood cell counts with an automated blood cell analyzer (Novobiotec, Beijing).

### Flow Cytometry Analysis

Mouse bone marrow and spleen tissues were collected, mechanically dissociated, filtered through a 70 µm cell strainer, and subjected to red blood cell lysis. For flow cytometry, bone marrow neutrophils were identified by the phenotype CD45⁺ CD11b⁺ Ly6G⁺ CXCR2⁺ using antibodies against CD45‐FITC, CD11b‐Brilliant Violet 510, Ly6G‐APC, and CXCR2‐PE. Splenic GC B cells were defined as B220^−^ Fas⁺ GL7⁺ using antibodies against B220‐FITC, Fas‐APC, and GL7‐PE. Splenic plasma cells were identified as B220^−^ CD138⁺ using antibodies against B220‐FITC and CD138‐Brilliant Violet 421. The Zombie NIR Fixable Viability Kit (Biolegend, USA; Cat. 423 105) was used to distinguish live from dead cells. All antibodies were used according to the manufacturers’ instructions (Table S2, Supporting Information). Data acquisition was performed on a CytoFLEX flow cytometer (Beckman Coulter, USA) and analyzed using CytExpert (version 2.6, Beckman Coulter, USA).

### RT‐PCR

Total RNA was extracted from spleen tissue or splenic neutrophils using TRIzol reagent (Invitrogen, Thermo Fisher Scientific, USA; Cat. 15596026CN). The extracted RNA was then reverse transcribed into complementary DNA using the PrimeScript RT kit (Takara Biotechnology, Japan; Cat. RR014A) according to the manufacturer's instructions. Quantitative real‐time PCR (qRT‐PCR) was performed on an ABI Prism 7500 system (Thermo Fisher Scientific, USA) using PowerUp SYBR Green Master Mix (Applied Biosystems, Thermo Fisher Scientific, USA; Cat. A25742) for gene expression analysis. The relative expression levels of target genes were calculated using the 2^^−ΔΔCt^ method, with β‐actin serving as the internal reference gene. The sequences of the primers used are provided in Table S3, Supporting Information.

### Spleen Histological Analysis

Spleen tissues were fixed in 4% paraformaldehyde in formalin and subsequently embedded in paraffin according to standard protocols. Paraffin‐embedded tissue sections were deparaffinized and rehydrated before staining with hematoxylin and eosin following standard procedures. For immunohistochemistry, sections were incubated with a primary antibody against Ly6G (Biolegend, USA; Cat. 127 602), followed by the application of HRP‐conjugated anti‐Rat IgG (ZSGB‐BIO, China; Cat. ZB‐2307).

### Statistics

Statistical analyses were conducted in R (version 4.3.3) and GraphPad Prism (GraphPad Software, San Diego, CA, USA). All data were expressed as mean ± standard deviation or 95% confidence interval unless otherwise specified. Group comparisons were performed using Student's t test, Mann–Whitney U test, one‐way ANOVA, or Kruskal–Wallis test as appropriate, with multiple‐comparison correction applied when indicated. Correlations were assessed using Spearman's rank correlation. For the clinical trial, patients were randomized to telitacicept or tacrolimus treatment. The primary endpoints were the 24‐week change from baseline in MGFA‐QMG, MG‐QOL15r, and MG‐ADL. For each endpoint, a separate baseline‐adjusted analysis of covariance (ANCOVA) was fitted, with treatment as the fixed factor and the corresponding baseline score as the covariate. Patients were further stratified by baseline absolute ANC into low and high subgroups using the cohort median as a prespecified cutoff; this threshold was chosen a priori to balance subgroup sizes and because it approximates the midpoint of the adult reference range, thereby avoiding pathological extremes. Post hoc analyses extended the ANCOVA by adding the ANC–based subgroup and the treatment × ANC‐based subgroup interaction, and estimated adjusted within‐stratum treatment contrasts to compare improvement between regimens. Using MGFA‐QMG as the exemplar endpoint, the treatment × ANC‐based subgroup interaction effect was further characterized by Cohen's *f* ^2^ and the corresponding post hoc statistical power. Sensitivity analyses augmented the post hoc model by including the ANC‐based subgroup×baseline score interaction to assess the robustness of inferences. Detailed statistical outputs and sample sizes (*n*) are reported in the corresponding figure legends.

## Conflict of Interest

The authors declare no conflict of interest.

## Author Contributions

Z.Z., J.D., and M.Q. contributed equally to this work and share the first authorship. Z.Z., J.D., M.Q., X.C., and X.S.: Conducting experiments, analyzing data, and writing. J.D. and J.B.: Data collection and data curation. Z.Z., M.Q., X.C., and X.S.: Experimental design, review, and editing.

## Supporting information



Supporting Information

## Data Availability

The data that support the findings of this study are available from the corresponding author upon reasonable request.
