# Supplementary material for: Single‐Cell Immune Profiling Reveals Neutrophils Promote Myasthenia Gravis Exacerbation Through BAFF Secretion
Source: Adv Sci (Weinh). 2025 Sep 25;12(45):e09260. doi: 10.1002/advs.202509260 (PMC12677686; doi:10.1002/advs.202509260)
Supplement: Supplementary file 1 — Supporting Information [file ADVS-12-e09260-s001.docx]

Supporting Information

Single-Cell Immune Profiling Reveals Neutrophils Promote Myasthenia Gravis Exacerbation through BAFF Secretion

Zhaoxu Zhang, Jingya Dong, Mengyuan Qiu, Jie Bai, Xufeng Cheng, and Xiaodong Song*


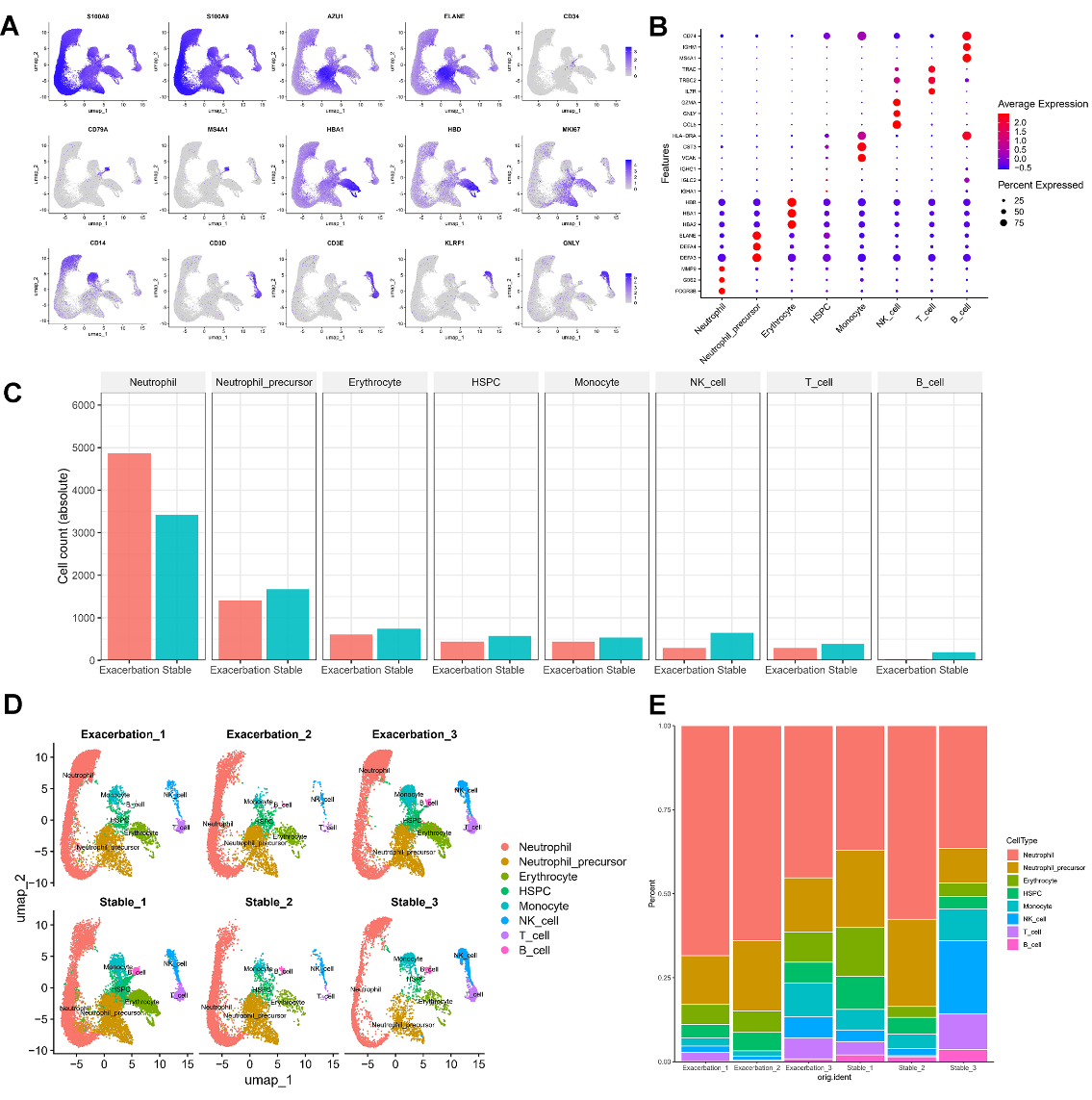


**Figure S1.** Characterization of bone marrow cell marker expression and cellular composition during the MG exacerbation phase.

(A) Feature plot displaying the expression patterns of representative markers in bone marrow cells. (B) Dot plots showing the expression levels of the top three marker genes for each cell type in the bone marrow. (C) The absolute numbers of bone marrow cells at the single-cell level were compared between the exacerbation group (*n* = 3) and the stable group (*n* = 3). (D) UMAP plot of bone marrow cells from individual donors (top three: exacerbation phase patients; bottom three: stable phase patients). (E) Relative changes in the cell-type ratios of bone marrow cells from individual donors (left three: exacerbation phase patients; right three: stable phase patients). Abbreviations: MG, myasthenia gravis; UMAP, uniform manifold approximation and projection.


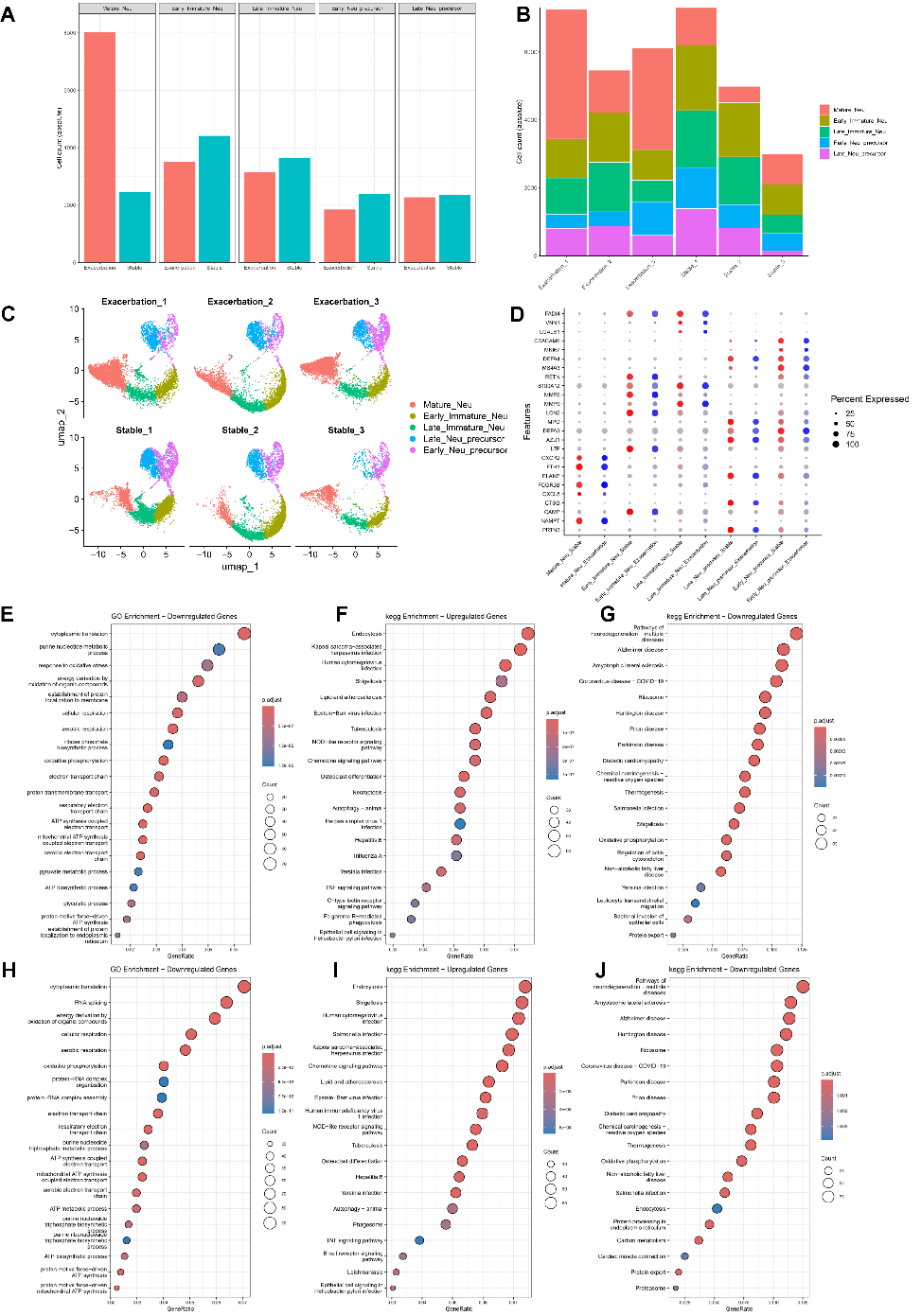


**Figure S2.** Bone marrow neutrophil subgroup analysis and functional enrichment.

(A) The absolute numbers of bone marrow neutrophil subgroups at the single-cell level were compared between the exacerbation group (n = 3) and the stable group (n = 3). (B) The absolute numbers of bone marrow neutrophil subgroups at the single-cell level from individual donors (left three: exacerbation phase patients; right three: stable phase patients). (C) UMAP plot of bone marrow neutrophils from individual donors (top three: exacerbation phase patients; bottom three: stable phase patients). (D) Dot plots displaying the top five marker gene expression profiles for different neutrophil subgroups between the MG exacerbation and stable groups. (E, H) GO enrichment bubble plots showing the top 20 GO terms of downregulated genes between mature neutrophils and immature neutrophils (mature neutrophils vs. late immature neutrophils in E, mature neutrophils vs. early immature neutrophils in H). (F, G, I, J) KEGG enrichment bubble plots showing the top 20 KEGG terms of upregulated (F, I) and downregulated (G, J) genes between mature neutrophils and immature neutrophils (mature neutrophils vs. late immature neutrophils in F and G; mature neutrophils vs. early immature neutrophils in I and J). Abbreviations: MG, myasthenia gravis; UMAP, uniform manifold approximation and projection; GO, Gene Ontology; KEGG, Kyoto Encyclopedia of Genes and Genomes.


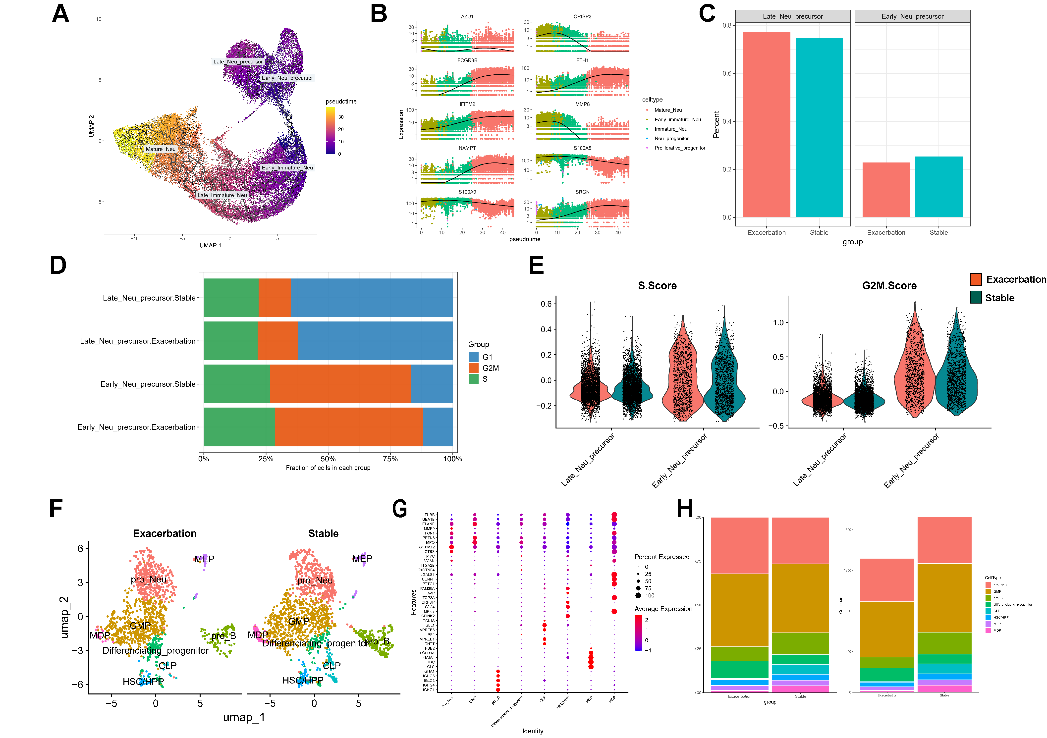


**Figure S3.** Neutrophil differentiation and HSPC subset composition in the MG exacerbation group.

(A) Inference of the pseudotime trajectories of bone marrow neutrophils. (B) Pseudotime expression dynamics of the top 10 genes across five neutrophil subpopulations. (C) The proportion of bone marrow neutrophil precursor subsets between the MG stable and exacerbation groups. (D) Proportions of cells in different cell cycle phases (G1, S, and G2/M) in neutrophil precursor subsets between the two groups. (E) S phase scores (left) and G2/M phase scores (right) of neutrophil precursor subsets between the two groups. (F) UMAP plot of bone marrow HSPC cells between exacerbation (left) and stable groups (right). (G) Dot plots showing the top five marker gene expression levels for different bone marrow HSPC subgroups. (G) The proportion of bone marrow HSPC subsets in the two groups. Abbreviations: MG, myasthenia gravis; UMAP, uniform manifold approximation and projection; HSPC, hematopoietic stem and progenitor cells.


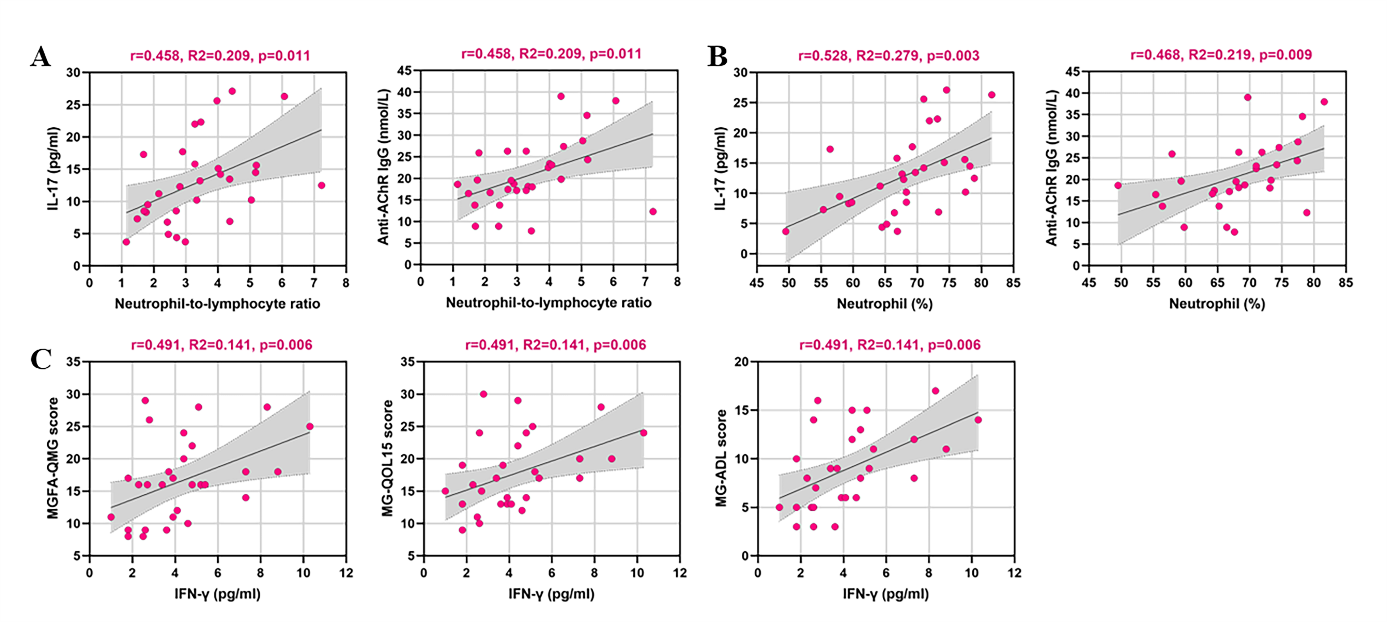


**Figure S4**. Correlation of neutrophil markers and inflammatory factors with MG disease severity

(A) Correlation between neutrophil-to-lymphocyte ratio (NLR) and serum IL-17 (left) and anti-AChR antibody levels (right). (B) Correlation between neutrophil proportions and serum IL-17 (left) and anti-AChR IgG levels (right). (C) Correlation between IFN-γ levels and MGFA-QMG (left), MG-QOL15r (middle), MG-ADL (right). Abbreviations: MG, myasthenia gravis; AChR, acetylcholine receptor; MGFA-QMG, Myasthenia Gravis Foundation of America Quantitative Myasthenia Gravis; MG-QOL15r, Myasthenia Gravis Quality of Life 15-item revised; MG-ADL, Myasthenia Gravis Activities of Daily Living.


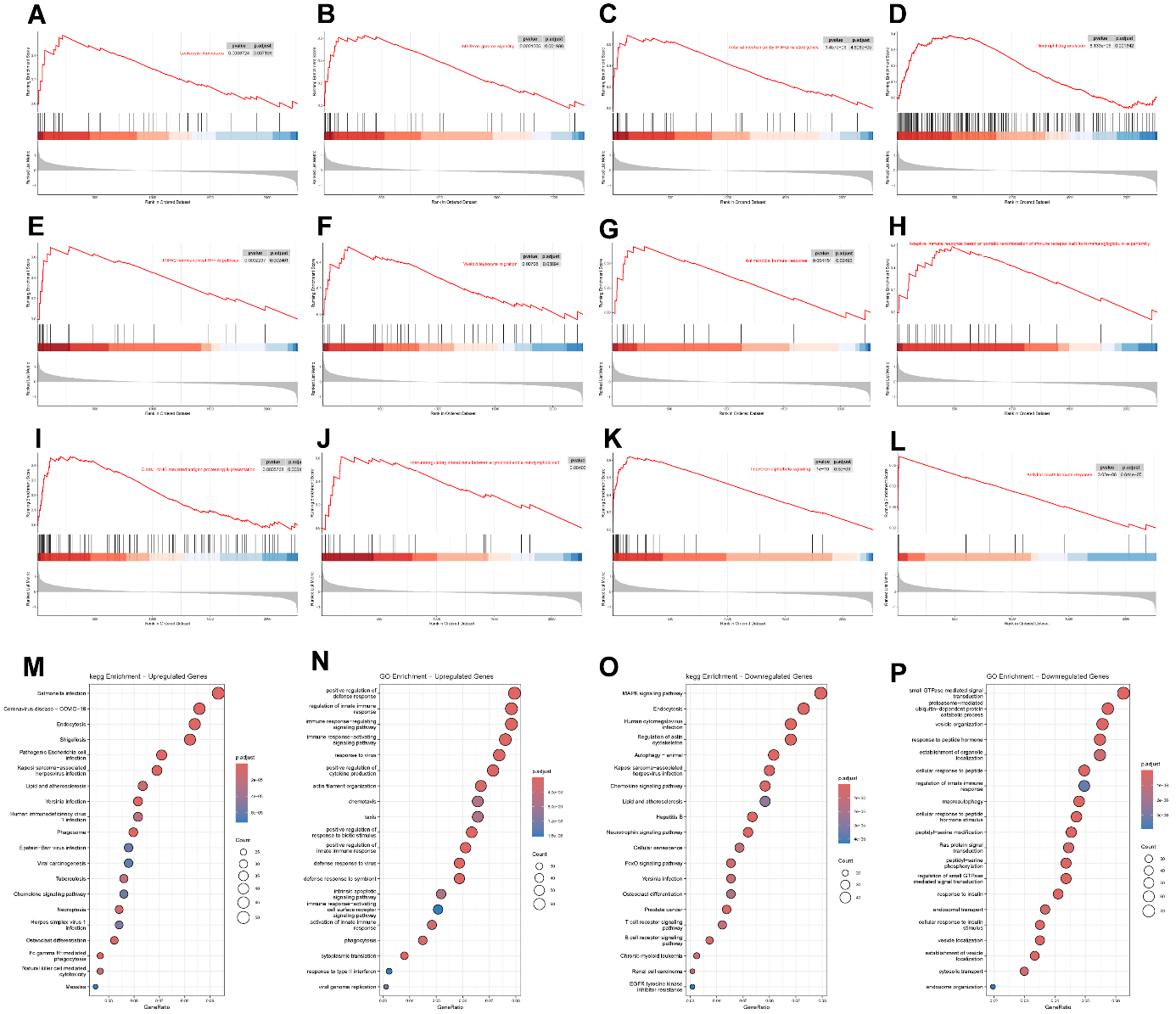


**Figure S5.** Enrichment analysis of DEGs in peripheral blood neutrophils in the MG exacerbation group.

(A-L) GSEA of upregulated DEGs in peripheral blood mature neutrophils between MG exacerbation and stable phases using Hallmark immune signature gene sets. (M-N) GO enrichment bubble plots showing the top 20 GO terms for downregulated (M) and upregulated (N) genes between peripheral blood mature neutrophils in the exacerbation and stable phases. (O-P) KEGG enrichment bubble plots showing the top 20 KEGG terms for downregulated (O) and upregulated (P) genes between peripheral blood mature neutrophils in the MG exacerbation and stable groups. Abbreviations: MG, myasthenia gravis; GSEA, Gene Set Enrichment Analysis; DEGs, differentially expressed genes; GO, Gene Ontology; KEGG, Kyoto Encyclopedia of Genes and Genomes.


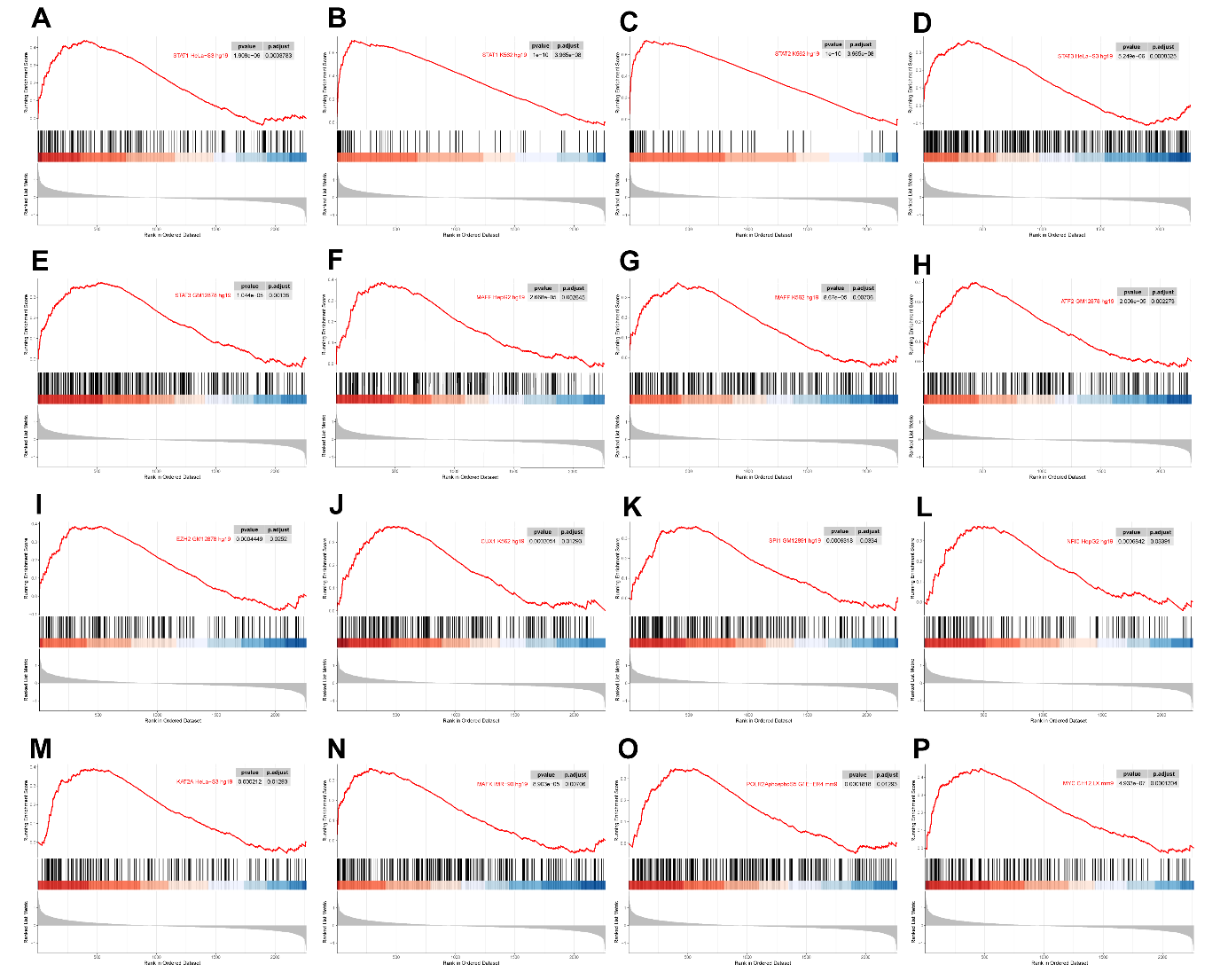


**Figure S6.** GSEA analysis of transcription factors in peripheral blood neutrophils of the MG exacerbation phase

(A-P) GSEA analysis of upregulated DEGs in peripheral blood mature neutrophils between MG exacerbation and stable phases using transcription factor-related gene sets. Abbreviations: GSEA, Gene Set Enrichment Analysis; DEGs, differentially expressed genes.


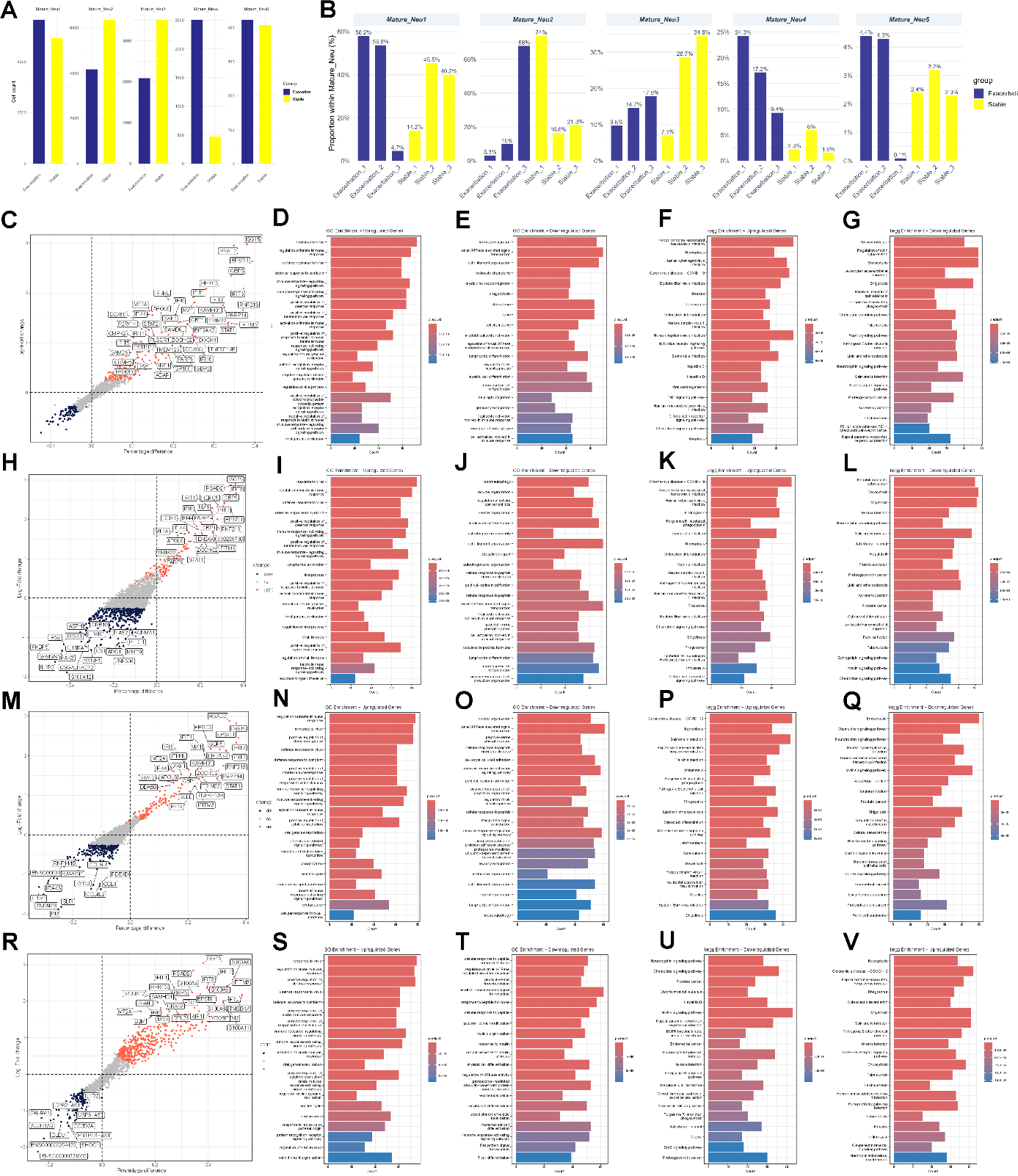


**Figure S7.** Differential expression and enrichment analysis of peripheral mature neutrophil subset 4 compared to other neutrophil subsets.

(A) The absolute numbers of peripheral neutrophil subgroups at the single-cell level were compared between the exacerbation group (*n* = 3) and the stable group (*n* = 3). (B) Relative changes in the cell-type ratios of peripheral neutrophil subgroups from individual donors (left three: exacerbation phase patients; right three: stable phase patients). (C, H, M, R) Volcano plots showing DEGs between mature neutrophil subset 4 and other mature neutrophil subsets, including mature neutrophil subset 1 (C), mature neutrophil subset 2 (H), mature neutrophil subset 3 (M), and mature neutrophil subset 5 (R). Red and blue dots indicate upregulated and downregulated genes in mature neutrophil subset 4, respectively. (D, E, I, J, N, O, S, T) GO enrichment bubble plots displaying the top 20 GO terms for upregulated (D, I, N, S) and downregulated (E, J, O, T) genes identified in the same comparisons. (F, G, K, L, P, Q, U, V) KEGG enrichment bubble plots showing the top 20 KEGG terms for upregulated (F, K, P, U) and downregulated (G, L, Q, V) genes in the corresponding DEG sets. Abbreviations: DEGs, differentially expressed genes; GO, Gene Ontology; KEGG, Kyoto Encyclopedia of Genes and Genomes.


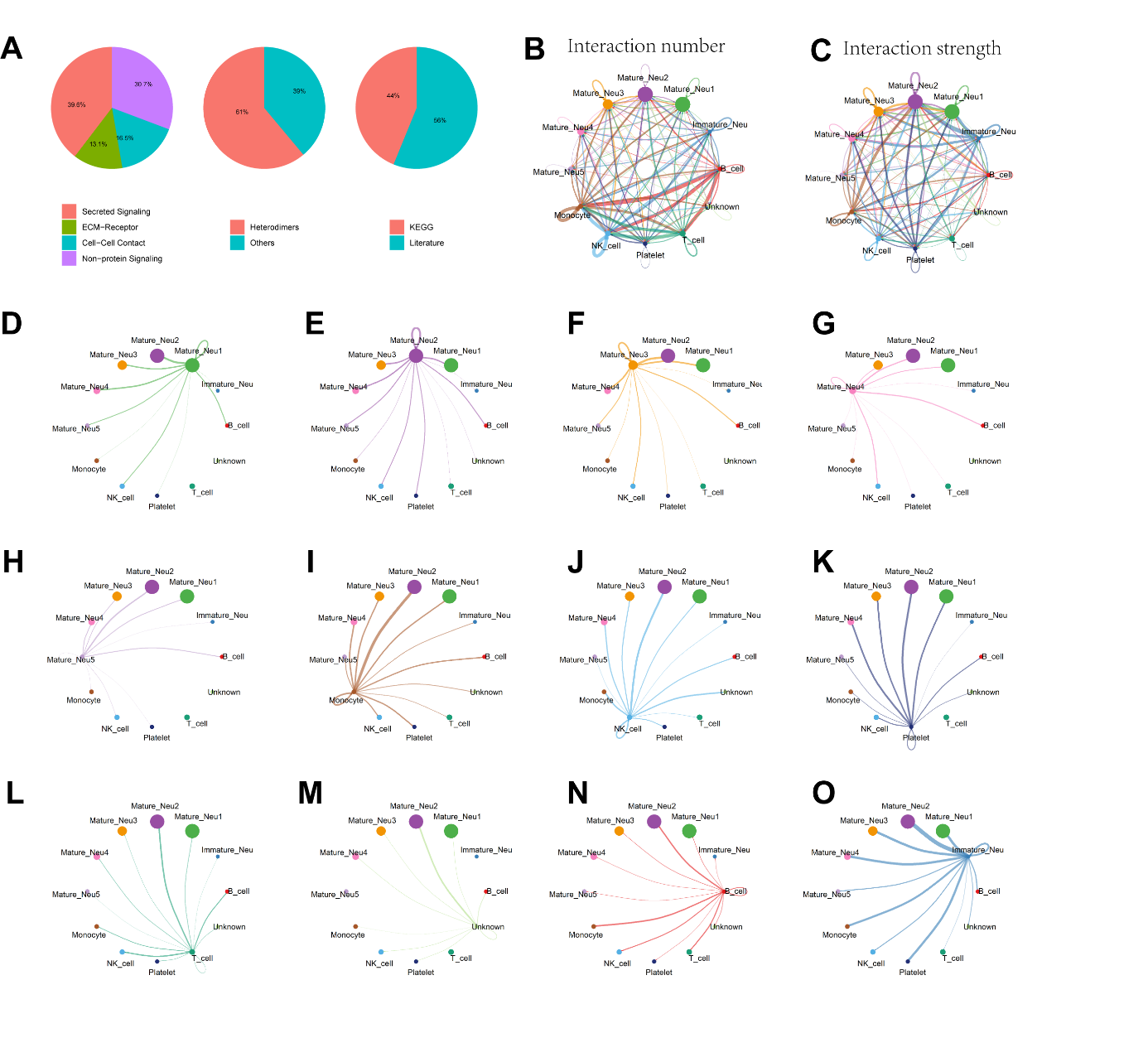


**Figure S8.** CellChat-based ligand-receptor interaction analysis in peripheral blood cells.

(A) Composition of ligand-receptor interaction types in the CellChat database, including secreted signaling, ECM-receptor interactions, cell-cell contact, non-protein signaling, heterodimers, and sources from KEGG and literature. (B, C) Circle plot showing ligand‒receptor interaction number and strength between different peripheral blood cell types. The number and strength of the ligand‒receptor interaction are represented by the line thickness. (D-O) Circle plots showing ligand-receptor communication networks, with each panel representing a different signal source cell type, including mature neutrophil subset 1 (D), mature neutrophil subset 2 (E), mature neutrophil subset 3 (F), mature neutrophil subset 4 (G), mature neutrophil subset 5 (H), monocyte (I), NK cell (J), platelet (K), T cell (L), unknown cell (M), B cell (N), and immature neutrophil (O), with other cell types as target cells. Abbreviations: KEGG, Kyoto Encyclopedia of Genes and Genomes; NK, natural killer.


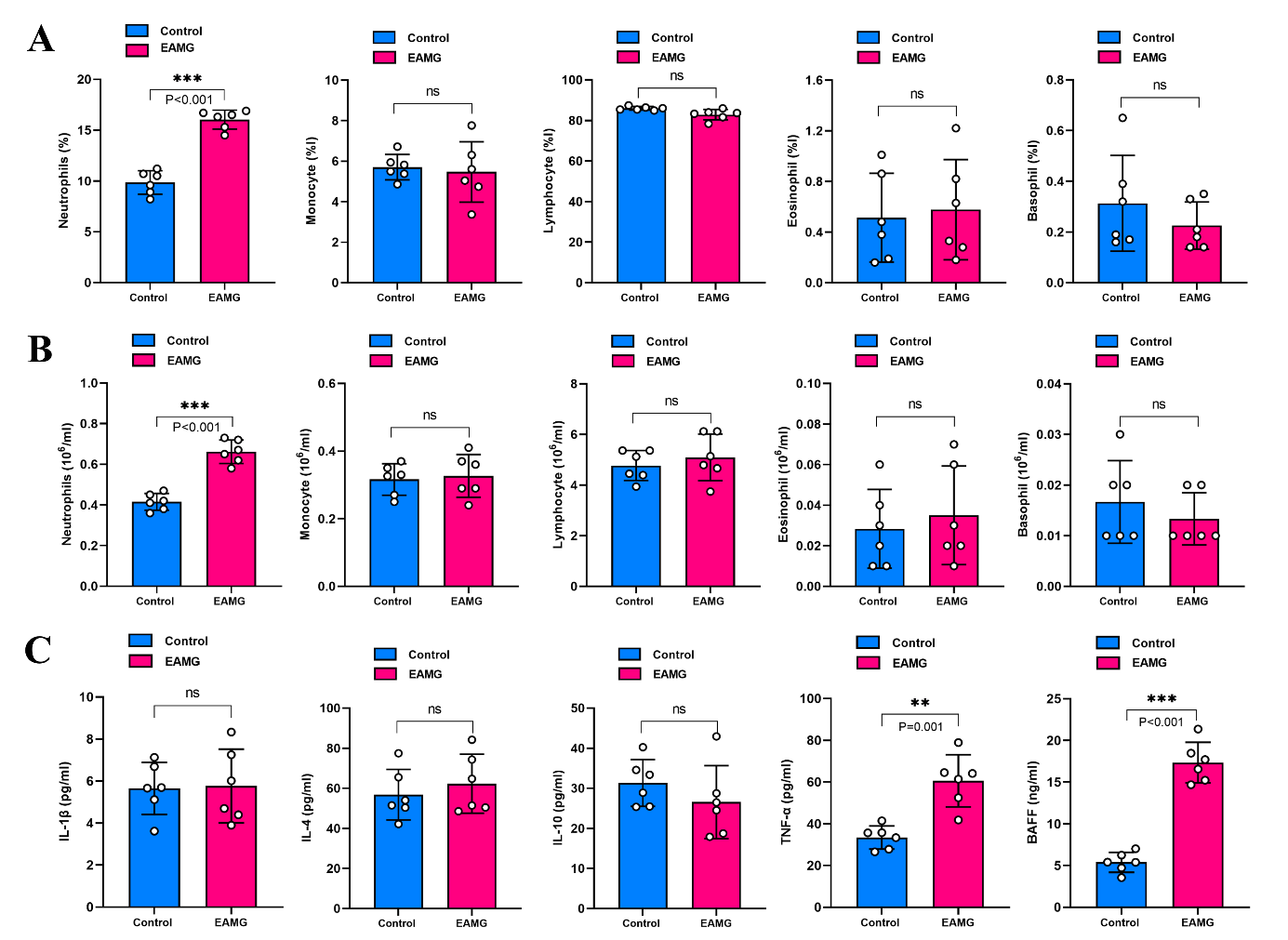


**Figure S9**. Comparative analysis of immune cell profiles and serum cytokines in control and EAMG groups

(A) Proportions of peripheral blood immune cells in control and EAMG groups. *n* = *6* per group. (B) Absolute counts of peripheral blood immune cells in control and EAMG groups. *n* = 6 per group. (C) Serum levels of IL-1β, IL-4, IL-6, IL-10, TNF-α, and BAFF in the two groups. *n* = 6 per group. Abbreviations: EAMG, experimental autoimmune myasthenia gravis.

Data are presented as mean ± 95% CI. ns, not significant; *P < 0.05; **P < 0.01; ***P < 0.001. P values were calculated using the Mann-Whitney U test. In all instances, *n* refers to the number in each group.


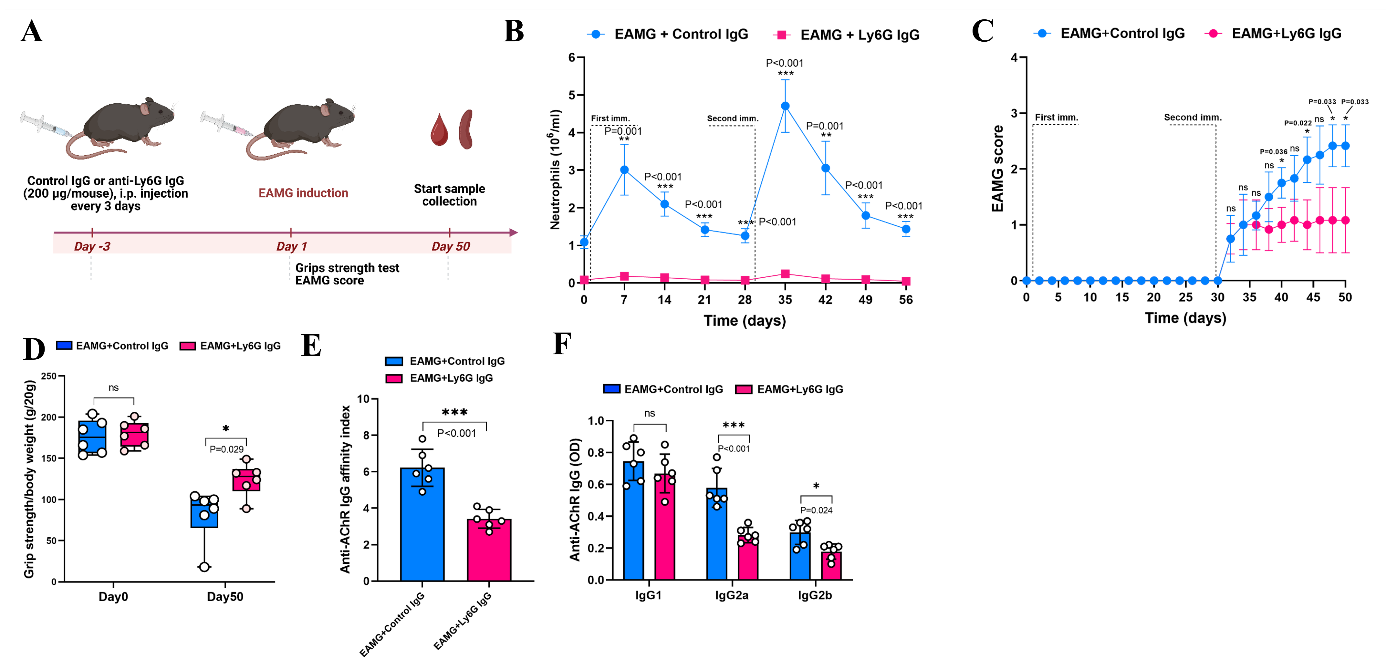


**Figure S10.** Impact of anti-Ly6G IgG treatment on clinical outcomes and anti-AChR IgG levels in EAMG mice

(A) Schematic diagram of the experimental protocol for anti-Ly6G IgG treatment in EAMG mice. (B) Analysis of peripheral blood neutrophil counts comparing changes between anti-Ly6G IgG-treated and control IgG-treated EAMG groups. *n* = 6 per group. (C, D) Evaluation of clinical disease severity after EAMG induction (C) and four-limb grip strength at 50 days post-induction (D), showing significant improvement in anti-Ly6G IgG-treated mice compared to controls. *n* = 6 per group. (E) Measurement of the anti-AChR IgG affinity index by ELISA at 50 days post-EAMG induction, expressed as the difference in OD between 450 nm and 630 nm wavelengths. *n* = 6 per group. (F) Quantification of anti-AChR IgG1, IgG2a, and IgG2b isotypes by ELISA at the same time point, with levels expressed as the difference in OD values between 450 nm and 630 nm wavelengths. *n* = 6 per group. Abbreviations: EAMG, experimental autoimmune myasthenia gravis; AChR, acetylcholine receptor; ELISA, enzyme-linked immunosorbent assay; OD, optical density.

Data are presented as mean ± 95% CI. ns, not significant; *P < 0.05; **P < 0.01; ***P < 0.001. P values were calculated using two-way ANOVA with Sidak’s post hoc analysis (B and C); Mann-Whitney U test with Sidak correction for multiple comparisons (D and F); or unpaired t test (E). In all cases, *n* refers to the number of samples per group.


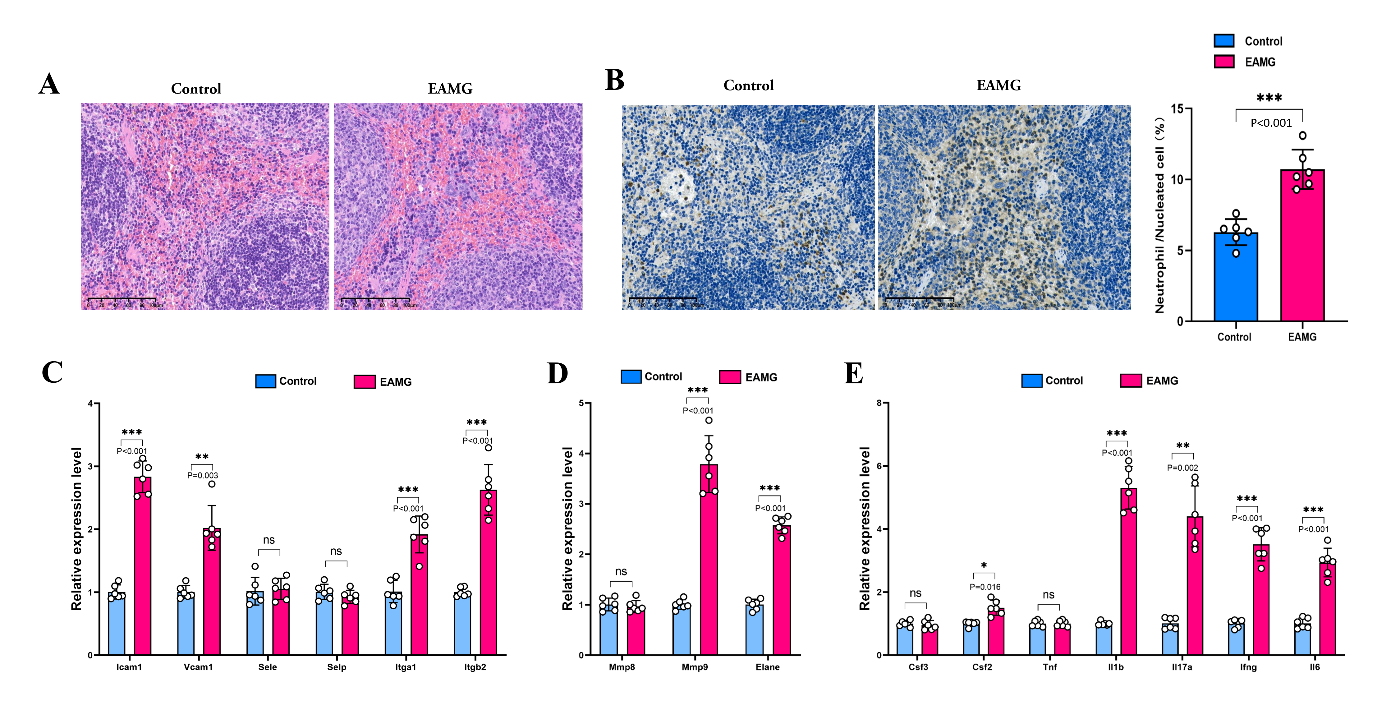


**Figure S11.** Splenic histopathology, neutrophil infiltration, and gene expression profiles in EAMG mice.

(A) HE staining of splenic tissue from control and EAMG groups, showing overall histopathological changes at 50 days post-EAMG induction. *n* = 6 per group. (B) Immunohistochemical analysis of Ly6G in spleen sections, demonstrating a significant increase in neutrophil percentage in the red pulp area of the spleen in the EAMG group at 50 days post-EAMG induction. *n* = 6 per group. (C-E) Quantitative PCR analysis of splenic tissue from EAMG mice, showing altered transcription levels of neutrophil adhesion molecules (Icam1, Vcam1, Pecam1, Sele, Selp, Itgal, and Itgb2) (C), matrix-degrading enzymes (Mmp8, Mmp9, and Elane) (D), and pro-inflammatory cytokines related to neutrophil chemotaxis (Csf3, Csf2, Tnf, Il1b, Il17a, Ifng, and Il6) (E) at 50 days post-EAMG induction. *n* = 6 per group.

Data are shown as mean ± 95% CI. ns, not significant; *P < 0.05; **P < 0.01; ***P < 0.001. P values were calculated using an unpaired t-test (B) or a Mann-Whitney U test with Sidak correction for multiple comparisons (C, D, and E). In all instances, *n* refers to the number in each group. Abbreviations: HE, hematoxylin and eosin; EAMG, experimental autoimmune myasthenia gravis.


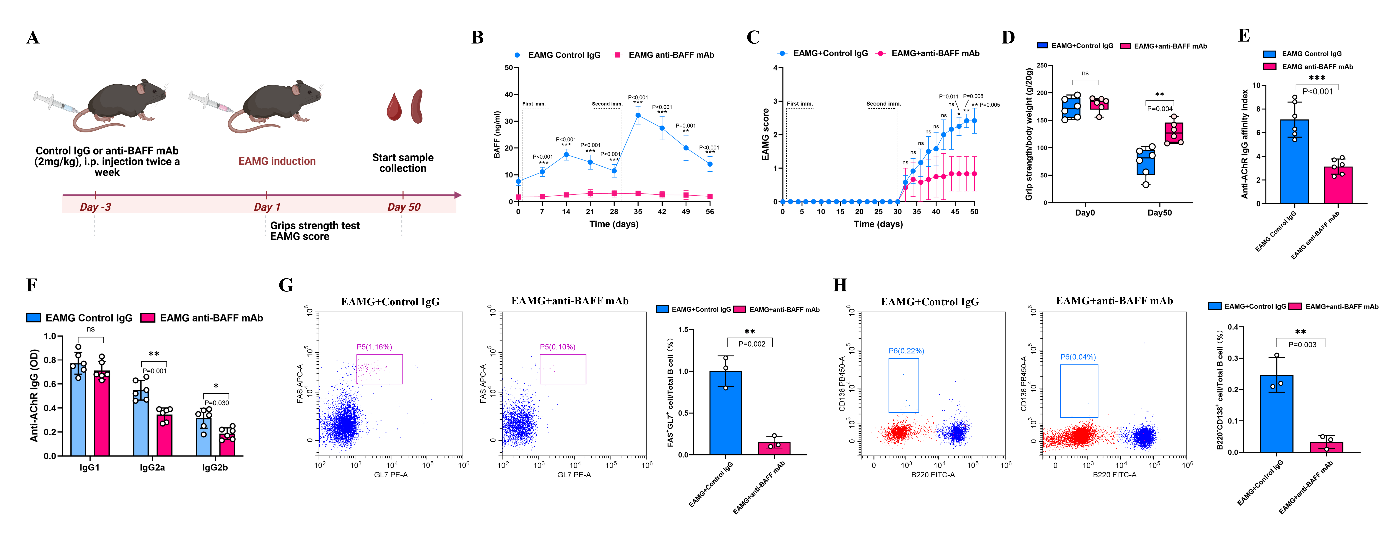


**Figure S12.** Therapeutic effects of BAFF blockade in EAMG.

(A) Experimental design of anti-BAFF mAb treatment in the EAMG model. (B) Dynamic changes in serum BAFF levels in EAMG mice receiving anti-BAFF mAb treatment after disease induction; n = 6 per group. (C–D) Mice receiving anti-BAFF mAb treatment showed significantly reduced EAMG scores (C) and improved four-limb grip strength (D) at day 50 compared to baseline and to controls; n = 6 per group. (E–F) At day 50 post-induction, the anti-BAFF mAb treatment group exhibited significantly reduced antibody affinity indices (E) and lower serum levels of anti-AChR IgG2a and IgG2b antibodies (F); n = 6 per group. (G–H) Flow cytometric analysis of splenic B220, CD138, Fas, and GL7 expression at day 50 post-induction showed significantly reduced proportions of germinal center B cells (Fas⁺GL7⁺) (G) and plasma cells (B220⁻CD138⁺) (H) in the anti-BAFF mAb treatment group compared to the Control IgG group; n = 6 per group. Abbreviations: EAMG, experimental autoimmune myasthenia gravis; BAFF, B-cell activating factor; AChR, acetylcholine receptor.

Data are presented as mean ± 95% CI. ns, not significant; *P < 0.05; **P < 0.01; ***P < 0.001. P values were calculated using two-way ANOVA with Sidak’s post hoc analysis (B and C); Mann-Whitney U test with Sidak correction for multiple comparisons (D and F); or unpaired t test (E, G, and H). In all cases, *n* refers to the number of samples per group.

**Supplementary Table 1: List of ELISA kits**

| **Target** | **ELISA kits** | **Company** | **Catalog number** |
| --- | --- | --- | --- |
| Human BAFF | BAFF human ProcartaPlex™ Simplex Kit | Invitrogen, Thermo Fisher Scientific | BMS2007INST |
| Human IL-2 | Human IL-2 Uncoated ELISA Kit | Invitrogen, Thermo Fisher Scientific | 88-7025-88 |
| Human IL-4 | Human IL-4 ELISA Kit | Invitrogen, Thermo Fisher Scientific | BMS225-2 |
| Human IL-6 | Human IL-6 ELISA Kit | Invitrogen, Thermo Fisher Scientific | EH2IL6 |
| Human IL-8 | Human IL-8 Uncoated ELISA Kit | Invitrogen, Thermo Fisher Scientific | 88-8086-88 |
| Human IL-10 | Human IL-10 Uncoated ELISA Kit | Invitrogen, Thermo Fisher Scientific | 88-7106-88 |
| Human IL-17 | Human IL-17A ELISA Kit, High Sensitivity | Invitrogen, Thermo Fisher Scientific | BMS2017-2HS |
| Human IFN-γ | Human IFN gamma ELISA Kit | Invitrogen, Thermo Fisher Scientific | KHC4021 |
| Human TNF-α | Human TNF alpha Uncoated ELISA Kit | Invitrogen, Thermo Fisher Scientific | 88-7346-88 |
| Mouse BAFF | Mouse BAFF ELISA Kit | Invitrogen, Thermo Fisher Scientific | EEL082 |
| Mouse IL-1β | Mouse IL-1 beta Uncoated ELISA Kit | Invitrogen, Thermo Fisher Scientific | 88-7013A-88 |
| Mouse IL-2 | Mouse IL-2 ELISA Kit | Invitrogen, Thermo Fisher Scientific | BMS601 |
| Mouse IL-4 | Mouse IL-4 Uncoated ELISA Kit with Plates | Invitrogen, Thermo Fisher Scientific | 88-7044-22 |
| Mouse IL-6 | Mouse IL-6 Uncoated ELISA Kit | Invitrogen, Thermo Fisher Scientific | 88-7064-88 |
| Mouse IL-10 | Mouse IL-10 Uncoated ELISA Kit | Invitrogen, Thermo Fisher Scientific | 88-7105-88 |
| Mouse IL-17 | Mouse IL-17A Uncoated ELISA Kit | Invitrogen, Thermo Fisher Scientific | 88-7371-88 |
| Mouse IFN-γ | Mouse IFN gamma Uncoated ELISA Kit | Invitrogen, Thermo Fisher Scientific | 88-7314-88 |
| Mouse TNF-α | Mouse TNF alpha ELISA Kit | Invitrogen, Thermo Fisher Scientific | BMS607-3 |

**Supplementary Table 2: Antibodies for flow cytometry**

| **Antibody/Target** | **Channel/Flurochrome** | **Company** | **Catalog Number** |
| --- | --- | --- | --- |
| APC anti-mouse Ly-6G | APC | Biolegend | 127613 |
| Brilliant Violet 510™ anti-mouse/human CD11b | Brilliant Violet 510 | Biolegend | 101245 |
| FITC anti-mouse CD45 | FITC | Biolegend | 103107 |
| FITC anti-mouse/human CD45R/B220 | FITC | Biolegend | 103205 |
| Brilliant Violet 421™ anti-human CD138 (Syndecan-1) | Brilliant Violet 421 | Biolegend | 356515 |
| PE anti-MU/HU GL7 Antigen (T/B Cell Act. Marker) | PE | Biolegend | 144607 |
| PE anti-mouse CD182 (CXCR2) | PE | Biolegend | 149303 |
| APC anti-mouse CD95 (Fas) | APC | Biolegend | 152603 |
| Zombie NIR™ Fixable Viability Kit | APC/Cy7 | Biolegend | 423105 |

**Supplementary Table 3: Primers for PCR**

| **Target** | **Forward** | **Reverse** |
| --- | --- | --- |
| ICAM1 | TAATGTCTCCGAGGCCAGGA | CGAGCTTCAGAGGCAGGAAA |
| VCAM1 | TTGACATCTCCCCCGGATCT | TGGATTTGGCCCCCTCATTC |
| SELE | TTAGCTTGCATGGCTCAGCT | CGCTGCAGCTCATGTTCATC |
| SELP | CCCCTGGCAAGTGGAATGAT | TGGGTAGCAGGAGCAGGTAT |
| ITGA1 | ATGACGCTCTGCCAAACTCA | GACGGTGAGGCTGACATTGA |
| ITGB2 | GTTTGTGGACAAGACGGTGC | TGCCGACCTCTGTCTGAAAC |
| MMP8 | AAAGGCAGACAGTACTGGGC | ATGGCTTGGACACTCCTTGG |
| MMP9 | CAGACCAAGGGTACAGCCTG | ATACAGCGGGTACATGAGCG |
| ELANE | GCACTGGCCTCAGAGATTGT | AAATGACCTCCACGCCTCTG |
| CSF3 | GTTCCCCTGGTCACTGTCAG | CTTCCTCACTTGCTCCAGGG |
| CSF2 | GGCATTGTGGTCTACAGCCT | GGGCTTCTTTGATGGCCTCT |
| TNF | ATGGCCTCCCTCTCATCAGT | AAGGTACAACCCATCGGCTG |
| IL1B | GGGCTGCTTCCAAACCTTTG | AAGACACAGGTAGCTGCCAC |
| IL17A | TCTCCACCGCAATGAAGACC | AAAGTGAAGGGGCAGCTCTC |
| IFNG | GCAAGGCGAAAAAGGATGCA | CGACTCCTTTTCCGCTTCCT |
| IL6 | GCCTTCTTGGGACTGATGCT | AGCCTCCGACTTGTGAAGTG |
| β-actin | CATGTACGTTGCTATCCAGGC | CTCCTTAATGTCACGCACGAT |

**Supplementary Table 4: Baseline characteristics of MG patients in the tacrolimus and telitacicept groups**

|  | **Tacrolimus**  **(n = 25)** | **Telitacicept**  **(n = 23)** | **Ovarall**  **(n = 48)** |
| --- | --- | --- | --- |
| Gender, n (%) |  |  |  |
| Female | 10 (40.0%) | 9 (39.1%) | 19 (39.6%) |
| Male | 15 (60.0%) | 14 (60.9%) | 29 (60.4) |
| Age, years |  |  |  |
| Mean (SD) | 58.4 (14.2) | 59 (15.6) | 58.7 (14.7) |
| Median (min, max) | 63.0 (24.0, 82.0) | 60 (26.0, 80.0) | 61.5 (24.0, 82.0) |
| Thymectomy, n (%) |  |  |  |
| Yes | 6 (24.0%) | 4 (17.4%) | 10 (20.8%) |
| No | 19 (76.0%) | 19 (82.6%) | 38 (79.2%) |
| MGFA classification, n (%) |  |  |  |
| IIa | 2 (8.0%) | 2 (8.7%) | 4 (8.3%) |
| IIb | 10 (40.0%) | 7 (30.4%) | 17 (35.4%) |
| IIIa | 4 (16.0%) | 3 (13.0%) | 7 (14.6%) |
| IIIb | 7 (28.0%) | 7 (30.4%) | 14 (29.2%) |
| IVa | 2 (8.0%) | 4 (17.4%) | 6 (12.5%) |
| Immunosuppressant history, n (%) |  |  |  |
| Azathioprine | 10 (40.0%) | 8 (34.8%) | 18 (37.5%) |
| Mycophenolate mofetil | 6 (24.0%) | 8 (34.8%) | 14 (29.2%) |
| Methotrexate | 8 (32.0%) | 7 (30.4%) | 15 (31.3%) |
| None | 1 (4.0%) | 0 (0.0%) | 1 (2.1%) |
| Steroid use, n (%) | 24 (96.0%) | 23 (100.0%) | 47 (97.9%) |
| Other MG medication |  |  |  |
| PE | 3 (12.0%) | 2 (8.7%) | 5 (10.4%) |
| IVIG | 4 (16.0%) | 5 (21.7%) | 9 (18.8%) |
| Both | 4 (16.0%) | 2 (8.7%) | 6 (12.5%) |
| None | 14 (56.0%) | 14 (60.9%) | 28 (58.3%) |
| MGFA-QMG |  |  |  |
| Mean (SD) | 18.8 (4.8) | 18.5 (6.3) | 18.6 (5.5) |
| Median (min, max) | 17.0 (11.0, 27.0) | 18.0 (9.0, 29.0) | 18.0 (9.0, 29.0) |
| MG-QOL15r |  |  |  |
| Mean (SD) | 18.8 (4.7) | 18.3 (5.2) | 18.6 (4.9) |
| Median (min, max) | 18.0 (12.0, 28.0) | 19.0 (10.0, 28.0) | 18.0 (10.0, 28.0) |
| MG-ADL |  |  |  |
| Mean (SD) | 10.3 (2.7) | 9.9 (3.4) | 10.1 (3.1) |
| Median (min, max) | 11.0 (5.0, 14.0) | 11.0 (3.0, 14.0) | 11.0 (3.0, 14.0) |

Abbreviations: MG, myasthenia gravis; MGFA, Myasthenia Gravis Foundation of America; SD, standard deviation; PE, plasma exchange; IVIG, intravenous immunoglobulin; MGFA-QMG, Myasthenia Gravis Foundation of America Quantitative Myasthenia Gravis; MG-QOL15r, Myasthenia Gravis Quality of Life 15-item revised; MG-ADL, Myasthenia Gravis Activities of Daily Living.

**Supplementary Table 5:** **Improvement in clinical score at week 24 in the tacrolimus and telitacicept groups**

|  | **Tacrolimus (n=25)** | **Telitacicept (n=23)** | **Difference** | **P value*** |
| --- | --- | --- | --- | --- |
| Change from baseline | Least squares mean (95% CI) | | (95% CI) |  |
| MGFA-QMG | -10.3 (-11.5, -9.1) | -11.6 (-12.8, -10.4) | 1.3 (-0.4, 3.0) | 0.374 |
| MG-QOL15r | -10.6 (-11.7, -9.5) | -11.9 (-13.0, -10.8) | 1.3 (-0.3, 2.9) | 0.468 |
| MG-ADL | -6.3 (-6.9, -5.6) | -6.9 (-7.6, -6.3) | 0.7 (−0.3, 1.7) | 0.318 |

Abbreviations: MGFA-QMG, Myasthenia Gravis Foundation of America quantitative myasthenia gravis; MG-ADL, MG-Related Activities of Daily Living; MG-QOL15r, revised 15-item Myasthenia Gravis Quality of Life; CI, confidence interval.

*P values were calculated using ANCOVA, adjusted for baseline clinical scores, and further corrected using the Bonferroni method for multiple comparisons.

**Supplementary Table 6: Baseline characteristics of MG patients in the low-neutrophil and high-neutrophil groups**

|  | **Low-neutrophil group**  **(n = 23)** | **High neutrophil group**  **(n = 25)** |
| --- | --- | --- |
| Gender, n (%) |  |  |
| Female | 12 (52.2%) | 7 (28.0%) |
| Male | 11 (47.8%) | 18 (72.0%) |
| Age, years |  |  |
| Mean (SD) | 60.4 (14.8) | 57.1 (14.8) |
| Median (min, max) | 64.0 (26.0, 82.0) | 60 (24.0, 80.0) |
| Thymectomy, n (%) |  |  |
| Yes | 3 (13.0%) | 7 (28.0%) |
| No | 20 (87.0%) | 18 (72.0%) |
| MGFA classification, n (%) |  |  |
| IIa | 3 (13.0%) | 12 (4.0%) |
| IIb | 14 (60.9%) | 3 (12.0%) |
| IIIa | 2 (8.7%) | 5 (20.0%) |
| IIIb | 2 (8.7%) | 12 (48.0%) |
| IVa | 2 (8.7%) | 4 (16.0%) |
| Immunosuppressant history, n (%) |  |  |
| Azathioprine | 7 (30.4%) | 11 (44.0%) |
| Mycophenolate mofetil | 6 (26.1%) | 8 (32.0%) |
| Methotrexate | 9 (39.1%) | 6 (24.0%) |
| None | 1 (4.3%) | 0 (0.0%) |
| Steroid use, n (%) | 22 (95.7%) | 25 (100.0%) |
| Other MG medication |  |  |
| PE | 2 (8.7%) | 3 (12.0%) |
| IVIG | 4 (17.4%) | 5 (20.0%) |
| Both | 3 (13.0%) | 3 (12.0%) |
| None | 14 (60.9%) | 14 (56.0%) |
| MGFA-QMG |  |  |
| Mean (SD) | 15.5 (4.2) | 21.5 (5.1) |
| Median (min, max) | 16.0 (9.0, 28.0) | 23.0 (10.0, 29.0) |
| MG-QOL15r |  |  |
| Mean (SD) | 15.4 (3.4) | 21.5 (4.2) |
| Median (min, max) | 16.0 (10.0, 24.0) | 22.0 (12.0, 28.0) |
| MG-ADL |  |  |
| Mean (SD) | 8.3 (2.6) | 11.8 (2.3) |
| Median (min, max) | 9.0 (3.0, 13.0) | 13.0 (5.0, 14.0) |

Abbreviations: MG, myasthenia gravis; MGFA, Myasthenia Gravis Foundation of America; SD, standard deviation; PE, plasma exchange; IVIG, intravenous immunoglobulin; MGFA-QMG, Myasthenia Gravis Foundation of America Quantitative Myasthenia Gravis; MG-QOL15r, Myasthenia Gravis Quality of Life 15-item revised; MG-ADL, Myasthenia Gravis Activities of Daily Living.

**Supplementary Table 7:** **Improvement in clinical score at week 24** **in the tacrolimus and telitacicept groups by neutrophil-level subgroup**

|  | **Neutrophil level** | **Tacrolimus** | **Telitacicept** | **Difference** | **P value*** |
| --- | --- | --- | --- | --- | --- |
| Change from baseline |  | Least squares mean (95% CI) | | (95% CI) |  |
| MGFA-QMG | Low group | -12.3 (-13.8, -10.7) | -10.9 (-12.6, -9.2) | -1.4 (-3.0, 0.8) | 0.433 |
| MG-QOL15r | Low group | -12.2 (-13.7, -10.6) | -11.2 (-12.9, -9.6) | -0.9 (-2.8, 0.9) | 0.743 |
| MG-ADL | Low group | -7.3 (-8.2, -6.3) | -6.7 (-7.7, -5.7) | -0.6 (−1.8, 0.7) | 0.771 |
| MGFA-QMG | High group | -8.5 (-10.0, -6.9) | -12.3 (-13.9, -10.7) | 3.8 (1.7, 5.9) | 0.001 |
| MG-QOL15r | High group | -9.1 (-10.7, -7.6) | -12.6 (-14.1, -11.0) | 3.4 (1.7, 5.9) | 0.003 |
| MG-ADL | High group | -5.3 (-6.2, -4.4) | -7.2 (-8.1, -6.3) | 1.9 (0.7, 3.1) | 0.007 |

Abbreviations: MGFA-QMG, Myasthenia Gravis Foundation of America quantitative myasthenia gravis; MG-ADL, MG-Related Activities of Daily Living; MG-QOL15r, revised 15-item Myasthenia Gravis Quality of Life; CI, confidence interval.

*P values were calculated using ANCOVA, adjusting for baseline clinical scores, neutrophil-level subgroup, and the interaction between treatment group and neutrophil-level subgroup, with further correction for multiple comparisons using the Bonferroni method. In the high neutrophil subgroup, 13 patients received tacrolimus, and 12 received telitacicept; in the low neutrophil subgroup, 12 patients received tacrolimus, and 11 received telitacicept.

**Supplementary Table 8:** **Improvement in clinical score at week 24** **in the tacrolimus and telitacicept groups by neutrophil-level subgroup—Sensitivity analysis**

|  | **Neutrophil level** | **Tacrolimus** | **Telitacicept** | **Difference** | **P value*** |
| --- | --- | --- | --- | --- | --- |
| Change from baseline |  | Least squares mean (95% CI) | | (95% CI) |  |
| MGFA-QMG | Low group | -11.6 (-13.3, -10.0) | -10.2 (-11.9, -8.4) | -1.5 (-3.0, 0.1) | 0.333 |
| MG-QOL15r | Low group | -11.6 (-13.2, -10.1) | -10.2 (-12.3, -8.2) | -1.5 (-3.2, 0.3) | 0.333 |
| MG-ADL | Low group | -6.9 (-7.8, -6.0) | -6.2 (-7.2, -5.3) | -0.6 (2.0, -0.7) | 0.559 |
| MGFA-QMG | High group | -8.1 (-9.6, -6.5) | -11.9 (-13.5, -10.3) | 3.8 (1.7, 5.9) | 0.001 |
| MG-QOL15r | High group | -8.1 (-9.4, -6.8) | -12.1 (-13.7, -10.6) | 3.8 (1.8, 5.9) | 0.001 |
| MG-ADL | High group | -4.7 (-5.7, -3.8) | -6.8 (-7.7, -5.9) | 2.0 (0.7, 3.1) | 0.001 |

Abbreviations: MGFA-QMG, Myasthenia Gravis Foundation of America quantitative myasthenia gravis; MG-ADL, MG-Related Activities of Daily Living; MG-QOL15r, revised 15-item Myasthenia Gravis Quality of Life; CI, confidence interval.

*P values were calculated using ANCOVA, adjusting for baseline clinical scores, neutrophil-level subgroups, the interaction between neutrophil-level-based subgroups and baseline clinical scores, and the interaction between treatment group and neutrophil-level-based subgroups, with further correction for multiple comparisons using the Bonferroni method. In the high neutrophil subgroup, 13 patients received tacrolimus, and 12 received telitacicept; in the low neutrophil subgroup, 12 patients received tacrolimus, and 11 received telitacicept.
